# Acute and Subchronic Toxicity Study of Flavonoid Rich Extract of *Glycyrrhiza glabra* (GutGard®) in Sprague Dawley Rats

**DOI:** 10.1155/2022/8517603

**Published:** 2022-03-31

**Authors:** Ranjit M. Bhide, Bharathi Bethapudi, Nehru Sai Suresh Chalichem, Muruganantham Nithyanantham, Sasi Kumar Murugan, Deepak Mundkinajeddu

**Affiliations:** ^1^Indian Institute of Toxicology, Pune, India; ^2^R&D Center, Natural Remedies Private Limited, Bengaluru, Karnataka, India; ^3^International Regulatory Affairs, Natural Remedies Private Limited, Bengaluru, Karnataka, India

## Abstract

*Glycyrrhiza glabra* (*G. glabra*) is well known for its health benefits based on the traditional and current scientific evidence. The aim of the present study was to evaluate the safety of GutGard, a standardised-flavonoid rich extract of *G. glabra*. The study was designed to evaluate the acute and subchronic oral toxicity of GutGard in Sprague Dawley rats according to the procedures and methods of Organisation for Economic Cooperation and Development (OECD) test guidelines for acute and subchronic toxicity. A single dose of GutGard at 5000 mg/kg body weight did not produce treatment related clinical signs of toxicity or mortality in any of the animals tested during the 14-day observation period. Therefore, the median lethal dose was estimated to be more than 5000 mg/kg. A subchronic oral toxicity study for 90 days in rats at the dose levels of 250, 500, and 1000 mg/kg did not show any treatment related adverse clinical signs. The treated animals exhibited normal weight gain and comparable feed intake. Ophthalmoscope examination did not reveal any abnormalities. Further, GutGard administration in rats did not show any clinical evidence of toxicity with respect to urinalysis, haematology, and blood chemistry parameters. The relative organ weight of vital organs did not differ significantly as compared to control. Gross and histopathological findings did not show any remarkable and treatment related changes. Based on the current experimental study findings, the median lethal dose (LD50) of GutGard was found to be >5000 mg/kg b.wt and the no observed adverse effect level (NOAEL) was found to be 1000 mg/kg rat b.wt.

## 1. Introduction

Traditional herbal medicines are widely used around the globe. During the past few decades, there is an increase in development and assessment of herbal formulations for various ailments. Although herbal medicines are considered natural, inherently safe, and nontoxic, they are not completely free from possible toxic effects. Hence, it becomes necessary to ascertain the safety of herbal medicines through short- and long-term toxicological studies [1, 2].

*Glycyrrhiza glabra* Linn, belonging to family Leguminosae/Fabaceae, is one of the most extensively used medicinal herbs in traditional systems of medicine [3]. It is also called as licorice/liquorice, sweet wood, mulethi, and yashtimadhu [4]. In traditional medicine, it is considered as “the grandfather of herbs.” It is one of the important herbs of Ayurveda used as a “Rasayana” for the treatment of respiratory and digestive disorders [4]. Traditionally, liquorice has been recommended in dyspepsia as an anti-inflammatory agent during allergen reaction and also as a prophylactic agent for gastric and duodenal ulcers [5]. It also possesses antistress and anabolic activities. In the Siddha system of medicine, it is used as demulcent, expectorant, antitussive, laxative, and sweetener, and in the Chinese system of medicine it is mostly prescribed for ailments related to spleen, liver, and kidney [4].

GutGard is a standardised extract obtained from the roots of *Glycyrrhiza glabra*, which is rich in flavonoids with negligible glycyrrhizin content developed specifically to safely manage the symptoms of functional dyspepsia and to reduce the gastric load of *Helicobacter pylori* [6, 7]. It was reported that GutGard causes acceleration of both gastric emptying and gastrointestinal transit in rats which makes it as a potential prokinetic agent in the management of gastrointestinal disorders [8]. It also possesses antiulcer activity as it exhibited protective effects against pylorus ligation, cold-restraint stress, and indomethacin induced ulcer [9]. The research work by Chandrasekaran et al. concluded that GutGard was considered nonmutagenic and safe by using series of (*in vitro*) genotoxicity tests [10]. Considering the beneficial role of GutGard in promoting gut health, acute and subchronic oral toxicity studies in rats have been carried out, to establish the no observed adverse effect level (NOAEL) through the current study.

## 2. Materials and Methods

### 2.1. Test Substance

The investigational test substance GutGard® is a flavonoid rich, standardised root extract of *Glycyrrhiza glabra* developed by Natural Remedies Pvt. Ltd, Bengaluru, India. GutGard is standardised to ≥10% w/w total flavonoids by HPLC including ≥3.5% w/w of glabridin as reported by Chandrasekaran et al. [11].

### 2.2. Experimental Animals

In this study, Sprague Dawley rats were used, which were sourced from National Institute of Biosciences, Pune, India, and acclimatised for a period of 5 days. The rats were housed in polycarbonate cages having dimensions of 44 cm × 28 cm × 15 cm (5/sex/cage except for acute oral toxicity study) with bedding of paddy and animal room was independently provided with 10–15 air changes per hour with a maintained room temperature of 22 ± 3°C, relative humidity of 30–70%, and an artificial light and dark cycle of 12 hours each. Rodent feed (pellet with approximately 19.5% crude protein and 3000 kcal/kg of energy) sourced from Nutrivet Life Sciences, Pune, India, and water passed through reverse osmosis membrane, were provided *ad libitum*.

### 2.3. Acute Oral Toxicity Study

A preliminary sighting study was performed to evaluate the acute oral toxicity of GutGard in Sprague Dawley rats (8–12 weeks/198–205 g) as per OECD principles of good laboratory practice and recommendations of CPCSEA. The dose was selected based on the OECD guidelines for testing of chemicals (no. 423) [12]. GutGard was administered as a suspension in 0.1% aqueous carboxymethyl cellulose (CMC) to three female rats (fasted) which were divided in two groups, i.e., group I consisting of 1 rat and group II consisting of 2 rats, at 5000 mg/kg body weight as single oral dose in a sequential manner. The dose volume was kept as 10 mL/kg body weight. The rats were allowed for food after 3-4 hours (approx.) of the administration of test material. After dosing, all the animals were observed for clinical signs of toxicity and mortality, immediately (0 to 5 minutes), 5, 10, 30, and 60 minutes, and 2^nd^, 4^th^, and 6^th^ hours on the day of dosing, and twice daily thereafter for 14 days, approximately at the same time. Body weight was measured before fasting, prior to administration of the test drug, weekly thereafter, and at termination of study on day 15, to calculate the change in body weight. Necropsy was performed after carbon dioxide asphyxiation, for all the animals at the end of the study period, i.e., on day 15 along with macroscopic examination of all the orifices and cavities and the findings were recorded.

### 2.4. 14-Day Repeated Dose Oral Toxicity Study

A dose range finding study was performed to evaluate the potential toxicity of repeated administration of GutGard in Sprague Dawley rats (5–7 weeks/125–150 g) in order to select dose levels for 90-day subchronic toxicity study. The study was performed in accordance with OECD guideline no. 407 [13]. In this study, a total of 40 Sprague Dawley rats (20 males and 20 females) were used which were divided into 4 groups (0, 250, 500, and 1000 mg/kg) of five animals each, per sex. All the animals were administered with GutGard by oral gavage for a period of 14 days at the dose levels of 250, 500, and 1000 mg/kg b.w. A concurrent control group of rats received the vehicle, i.e., 0.1% aqueous carboxymethyl cellulose (CMC). All the animals were observed for mortality and clinical signs, twice daily throughout the study period of 14 days. Body weights were recorded on the day of randomisation, on the first day of treatment before dosing, days 4, 8, 11, and 14, and fasting body weight at scheduled sacrifice on day 15. Rats were examined once daily for clinical signs to assess the behavioural status of each animal. All the rats were sacrificed on day 15 by carbon dioxide induced asphyxia and gross lesions were recorded.

### 2.5. 90-Day Repeated Dose Subchronic Oral Toxicity Study

#### 2.5.1. Doses and Treatment

A 90-day repeated dose oral toxicity study was conducted to assess toxicological profile of GutGard, i.e., to determine any possibility of toxic influence on various organs and NOAEL in rats after 90 days of administration. The study was performed in accordance with the OECD test guideline no. 408 (OECD, 1998) [14]. 50 male and 50 female rats (5–7 weeks/95–114 g) were randomised into four groups with 10 animals/sex for main groups (0, 250, 500, and 1000 mg/kg) and 5 animals/sex/for reversal groups (0, 1000 mg/kg). Reversal groups were included to study the reversibility/delayed occurrence of symptoms. The control animals were administered with vehicle 0.1% aqueous carboxymethyl cellulose only.

All the animal experiments were conducted in accordance with the ethical regulations of Committee for the Purpose of Control and Supervision of Experiments on Animals (CPCSEA), India, and in compliance with the good laboratory practices (GLP) and also ensured that all animal experiments were complied with the ARRIVE guidelines. All the experimental procedures were approved by Institutional Animal Ethical committee of Indian Institute of Toxicology, Pune, India (approval numbers: 16576, 16577, and 16578).

#### 2.5.2. Body Weight and Feed Consumption

Body weights of all the animals were recorded on the day of administration, on the first day of treatment before dosing, weekly thereafter, and a fasting body weight at scheduled sacrifice on day 91 and day 119. The quantity of feed consumed by control and different treatment groups was recorded on commencement of treatment and weekly thereafter until scheduled sacrifice.

#### 2.5.3. Ophthalmologic Examination

Eyes of all the animals were examined prior to the initiation of the dosing and at scheduled sacrifice. Eye examination was carried out using a HEINE mini 2000 ophthalmoscope, Germany, after the induction of mydriasis with 1% solution of tropicamide sulphate solution.

#### 2.5.4. Clinical Observations and General Appearance

Rats were examined once daily for clinical signs and detailed clinical observations were made to assess the behavioural status of each animal, at the initiation of the study followed by weekly intervals. Detailed clinical observations include home cage observations, handling observations, and open field observations. In home cage observations, rats were checked for behaviour, alterations, vocalizations, respiration, and palpebral closure. Handling observations include reaction to removal, reaction to handling, urination, defecation, prominence of eye, lacrimation, salivation, piloerection, examination of mucus membrane, examination of skin/fur, examination of natural orifices, and animal appearance. On the other hand, open field observations include stereotype behaviour, bizarre behaviour, rearing (rears), clonic and tonic movements, gait pattern, mobility score, severity of gait, and pupillary response.

#### 2.5.5. Functional Observations

Towards the end of exposure period of 90 days and recovery period of 118 days, sensory reactivity to stimuli of different types (e.g., auditory, visual, and proprioceptive stimuli) was assessed for all animals. Grip strength of fore limbs was measured with a digital grip strength meter (Columbus Instruments International Corporation, Ohio, USA) to determine the ability of the rat of grasping and holding on the mesh platform. Motor activity of each animal was monitored using an automated animal activity measuring system (Columbus Instruments, Ohio, USA).

#### 2.5.6. Clinical Pathology

Haematological and clinical biochemistry investigations were performed on day 91 (control and treated groups) and day 119 (reversal groups) [15, 16]. All the rats were subjected for overnight fasting and blood samples were withdrawn from orbital sinus and collected into tubes containing potassium EDTA (100 *μ*l of 1.5 mg/mL), sodium heparin (100 *μ*l of 25 IU/mL), and sodium citrate (100 *μ*l of 3.8% solution per mL of blood). The blood samples collected within the heparinised tubes were centrifuged at 3000 rpm for 10 mins in order to separate the plasma.

EDTA tubes were used for the estimation of haematological parameters using Beckman Coulter haematology analyser, USA. It includes haemoglobin, red blood corpuscles, haematocrit, mean corpuscular volume, and mean corpuscular haemoglobin concentration, platelets, and white blood corpuscles.

Tubes with sodium heparin were used for clinical chemistry estimations using Dimension Xpand^Plus^, VeTEX (Veterinary Chemistry Expert), and Clinical Chemistry Autoanalyser system, USA, which include total protein, blood urea nitrogen, urea, alanine aminotransferase, aspartate aminotransferase, alkaline phosphatase, gamma glutamyl transferase, glucose, calcium, phosphorus, albumin, total bilirubin, creatinine, total cholesterol, triglycerides, sodium, potassium, and chloride. On the other hand, sodium citrate tubes were used for the estimation of prothrombin time. All biochemical investigations were conducted immediately after the collection of blood samples using flex reagent cartridge supplied by SIEMENS (Siemens Healthcare Diagnostics Inc., USA).

All the collected blood samples were placed on the ice tray and transferred to the clinical chemistry department. The blood samples were centrifuged to get plasma sample which were kept in refrigerator (temperature between 2°C and 8°C) till processing on the same day of necropsy.

#### 2.5.7. Urinalysis

The urine samples were collected on day 91 from main groups and on day 119 from reversal groups by placing the animals in metabolic cages for 16 hours and analysed for physical properties such as volume, appearance, and colour and chemical properties like pH, specific gravity, glucose, ketones, bilirubin, urobilinogen, occult blood, and nitrite, using Multistix, Siemens Healthineers, India.

#### 2.5.8. Gross Pathology and Organ Weights

After 90 days of oral administration, all surviving rats were sacrificed on day 91, whereas rats from recovery groups were sacrificed on day 119 by CO_2_ asphyxiation and observed for gross pathology and then subjected to complete necropsy. Liver, kidneys, adrenal glands, epididymides/uterus, thymus, spleen, brain, heart, and ovaries/testes were dissected free of fat and weighed. The paired organs were weighed together.

#### 2.5.9. Histopathology

All the tissues were preserved in 10% neutral buffered formalin whereas eyes and tests were preserved in Davidson's solution for 24 hours and transferred to 10% neutral buffered formalin. Animals from control and the highest dose level of 1000 mg/kg were subjected to histopathological examination: adrenals, aorta, brain (cerebrum, cerebellum, and pons), caecum, colon, duodenum, epididymides, eyes, heart, ileum, jejunum, kidneys, liver, lungs, mesenteric lymph nodes, muscles-skeletal muscle, oesophagus, ovaries, pancreas, pharyngeal lymph nodes, pituitary, prostate, rectum, salivary gland, sciatic nerve, seminal vesicles, skin with mammary gland, spleen, spinal cord (cervical, mid-thoracic, and lumbar), sternum with bone marrow, stomach, testes, thymus, thyroid/parathyroid, trachea, urinary bladder, and uterus. The organs/tissues showing abnormality in animals from high dose group were subjected for histopathological examination from lower dose groups.

## 3. Statistical Analysis

All quantitative data were expressed in mean and standard deviation. All the parameters characterized by continuous data were subjected to Bartlett's test to meet the homogeneity of variance before conducting Analysis of Variance (one-way ANOVA) and Dunnett's *t*-test. Although the data did not meet the homogeneity of variance, Student's *t*-test was performed to calculate significance. The variance was evaluated at 1% as well as 5% level of significance.

## 4. Results

### 4.1. Acute Oral Toxicity Study

Animals in both groups, treated at a dose level of 5000 mg/kg, have survived throughout the study at a period of 14 days without any signs of toxicity. The percent body weight gain after 7 and 14 days was found to be 6.13% and 12.41% in group I animal whereas in group II it was found to be 6.28% and 12.14%, respectively, and mean of three animals is represented in Table 1. Since the gross pathological examination did not reveal any abnormalities, histopathology was not performed.

### 4.2. 14-Day Repeated Dose Oral Toxicity Study

All animals from control and different dose levels survived throughout the study period with no clinical signs of toxicity and exhibited normal body weight gain throughout the dosing period of 14 days (Table 2). Before and after commencement of treatment, rats from all treated groups and control group revealed normal behaviour, alterations, and respiration in home cage observations. N one of the animals has showed any abnormality during handling observations and in an open field observation. Gross pathological examination revealed slightly enlarged spleen in four male and three female animals from 1000 mg/kg dose group whereas in animals from control, 250 and 500 dose groups did not reveal any abnormality.

### 4.3. 90-Day Repeated Dose Oral Toxicity Study

#### 4.3.1. Body Weight Gain and Food Consumption

**Figure 1:**
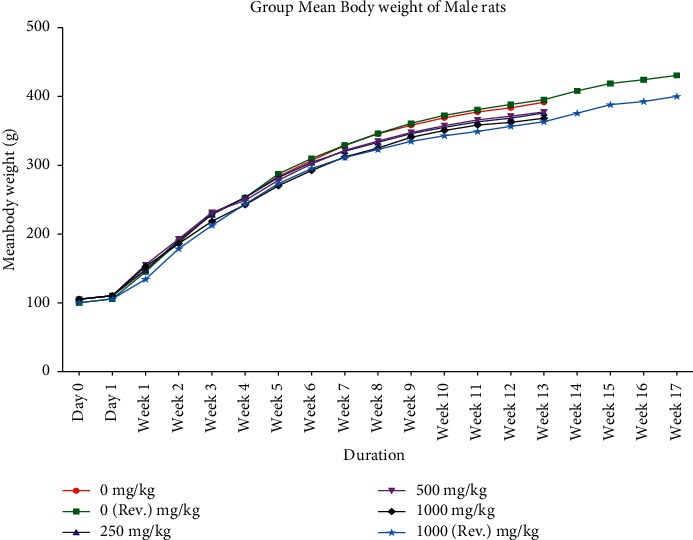
Group mean body weight (mean ± SD) of male rats orally administered with GutGard for 90 days (*n* = 10; *n* = 5 for reversal groups; *p* ≤ 0.05).

**Figure 2:**
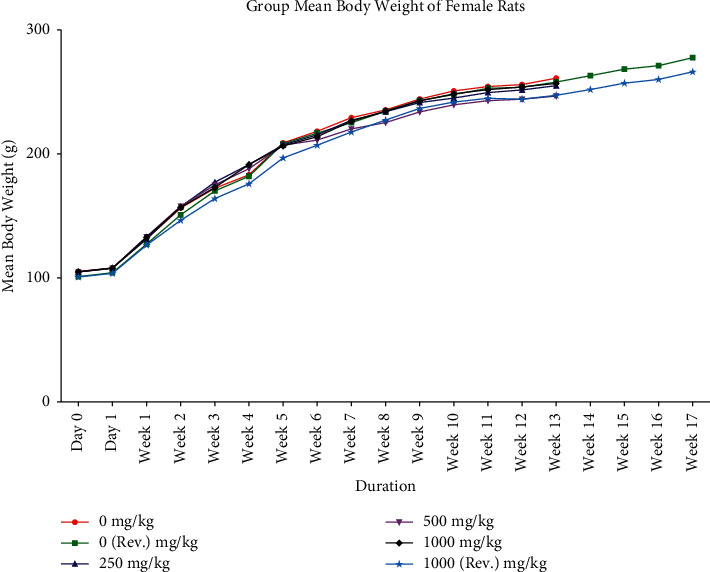
Group mean body weight (mean ± SD) of female rats orally administered with GutGard for 90 days (*n* = 10; *n* = 5 for reversal groups; *p* ≤ 0.05).

**Figure 3:**
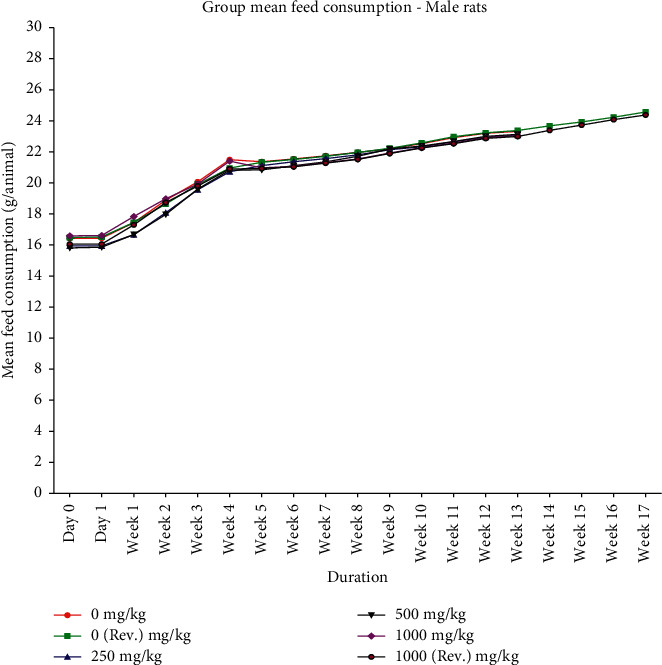
Group mean feed consumption (mean ± SD) of male rats orally administered with GutGard for 90 days (*n* = 10; *n* = 5 for reversal groups; *p* ≤ 0.05).

**Figure 4:**
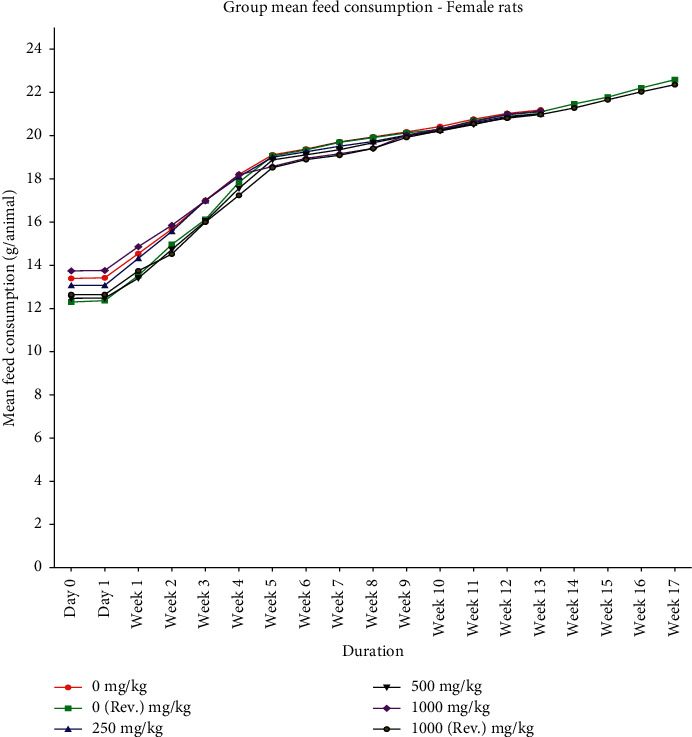
Group mean feed consumption (mean ± SD) of female rats orally administered with GutGard for 90 days (*n* = 10; *n* = 5 for reversal groups; *p* ≤ 0.05).

Animals from control and treated groups exhibited normal body weight gain throughout the dosing period of 90 days and after dosing recovery period (Figures 1 and 2). With respect to feed consumption, no difference was observed in control, all treated dose groups, and reversal dose groups at different evaluation time intervals (Figures 3 and 4).

#### 4.3.2. Ophthalmologic Examination

No ocular abnormalities were observed in the ophthalmological examination of control and all the treated dose group rats.

#### 4.3.3. Clinical Observations and General Appearance

Animals from either main or reversal groups did not show any clinical signs of toxicity throughout the dosing period of 90 days and during the postdosing recovery period.

Detailed clinical observations which include home cage observation, handling observation, and open field observations of rats from all treated and control groups did not reveal any abnormalities. All animals showed normal arousal level, visual response, touch response, auditory response, tail pinch response, visual replacing response, and air righting reflex. Mean values of grip strength and motor activity of male and female rats from all treated dose groups were comparable to control groups.

#### 4.3.4. Haematological Investigations

Few haematological investigations were found to be statistically significant when compared with those of respective controls. In male rats, significant increase in HCT values was observed in 500 mg/kg dose group whereas 1000 mg/kg dose group showed significant increase in MCV, MCH, and platelets values on day 91. Male rats of 250 mg/kg and 1000 mg/kg and female rats of 1000 mg/kg dose group showed increase in total WBC count on day 91 (Tables 3 and 4). However, dose dependency was not observed in the occurring events.

#### 4.3.5. Clinical Biochemistry Investigations

Biochemical investigations showed elevated levels of creatinine in 250 mg/kg and 500 mg/kg dose groups and chloride in 500 mg/kg dose groups of male rats whereas a decrease in levels of potassium in 250 mg/kg was observed in comparison to respective control and was found to be clinically nonsignificant. A decrease in the levels of calcium and sodium in 1000 mg/kg reversal groups in relation to respective control reversal group was observed. In female rats, levels of total protein, calcium, phosphorus, and albumin were found to be decreased in 500 mg/kg dose group and 250 mg/kg dose group showed decrease in phosphorus and total cholesterol levels whereas a decrease in phosphorus and sodium levels was observed in 1000 mg/kg dose group. All these changes were compared statistically with their respective control; i.e., 250, 500, and 1000 mg/kg were compared with control group whereas 1000 mg/kg reversal group was compared with control reversal group (Tables 5 and 6).

#### 4.3.6. Urinalysis

At the end of the dosing period, i.e., in weeks 13 and 17 (on days 86, 87, 88, and 119), urine analyses were conducted and found to have lack of significant variations between treated groups and respective control groups (Tables 7 and 8).

#### 4.3.7. Gross Pathology and Organ Weights

On day 91, organ weight of male animals of 1000 mg/kg dose group showed increased relative weights of liver and kidneys whereas 500 mg/kg and 1000 mg/kg dose groups showed increased relative weight of adrenals (Tables 9–12). At the end of postdosing recovery period, i.e., on day 119, organ weight of animals of 1000 mg/kg reversal group was found to be comparable with that of respective control reversal group (Table 10).

In comparison with controls on day 91, female animals of 500 mg/kg and 1000 mg/kg dose groups showed increased relative weight of liver, whereas 500 mg/kg dose group alone has shown increased relative weight of kidneys. Increase in relative weights of heart was observed with 250 mg/kg, 500 mg/kg, and 1000 mg/kg dose groups. The 1000 mg/kg reversal group showed decrease in the relative weight of spleen at the end of postdosing recovery period on day 119 when compared to its respective control reversal group (Table 12).

Gross pathological examination of male and female animals from control and different treatment groups did not reveal any abnormality.

### 4.3.8. Histopathology

Histopathological examination showed minimal, focal to multifocal mononuclear cells infiltration of liver and kidneys along with inconsiderable dilatation of kidneys. The minimal, focal to multifocal alveolar haemorrhages in the lungs were also observed as well as minimal, focal vacuolation and/or cysts in the adrenals and pituitary. Histopathological examination also showed the minimal, diffuse dilation in the uterus and minimal, luminal seminal coagulum in urinary bladder along with minimal, multifocal hemosiderosis in spleen. All these observed changes were found irrespectively of sex in both control and high dose groups. The changes observed in the control and high dose treatment groups are comparable and hence are considered as incidental (Supplementary Figure 1).

## 5. Discussion

Hippocrates, the father of modern medicine said, “All disease begins in the gut,” indicating the importance of gut health in overall wellbeing [17]. The term “gut health” includes effective digestion and absorption of food, the absence of gastrointestinal illness, normal and stable intestinal microbiota, effective immune status, and state of wellbeing [18]. Research over the last few decades has revealed that the health of the gut has a huge impact on overall health and an unhealthy gut can contribute to a wide range of health issues, like constipation, irritable bowel syndrome, inflammatory bowel diseases, and functional dyspepsia [19]. Gastrointestinal disorders not only affect the quality of life of an individual but also have significant costs. For hundreds of years, ancient herbal remedies have been used for treatment of gut dysfunction. GutGard is one such herbal extract, standardised to contain flavonoids derived from *Glycyrrhiza glabra*, which supports in maintaining healthy gastrointestinal tract. However, herbal formulations are perceived to be naturally safe but there is a lack of scientific evidence on the adverse effects of herbal formulations. Therefore, in this study, toxicological investigation on GutGard was conducted to understand the no observed adverse effect level (NOAEL) in Sprague Dawley rats.

In order to establish the safety of test substance, acute oral toxicity is considered as the preliminary step, as it provides information on health hazards that may arise from acute exposure. From the current study, it was clearly evident that the single acute oral dose of GutGard administered to Sprague Dawley rats at the dose level of 5000 mg/kg could not cause any mortality and hence it was found to be safe on acute exposure. Any test substance with LD50 greater than 5000 mg/kg rat body weight is considered as highly safe and unclassified as per the GHS 5 system of classification [20].

Repeated dose toxicity studies are conducted to evaluate the possible adverse effects that may likely arise from repeated exposure over a prolonged period of time. It also provides information about possibilities of cumulative effects and an estimate of the dose at which there is no observed adverse effect on continuous exposure of the test substance. As per regulatory guidelines for subchronic toxicity testing, 14-day repeated oral toxicity study was performed in the current experiment to select the dose levels for 90-day oral toxicity study. GutGard on administration at dose levels of 250, 500, and 1000 mg/kg b.w. did not reveal any mortality, unnatural weight gain, and abnormalities in clinical and behavioural signs, whereas gross pathological examination showed slight enlargement of spleen in 1000 mg/kg group (only) which was found to be not dependent on dose and lacks any biological significance due to the absence of any clinical signs of toxicity and hence was considered to be of no toxicological significance.

On the basis of findings from 14-day dose range study, dose levels of 250, 500, and 1000 mg/kg b.w. were selected for 90-day subchronic oral toxicity study in rats. Oral administration of GutGard up to the dose level of 1000 mg/kg did not show mortality or any clinical signs of toxicity that affects the homeostasis of the biological systems, throughout the dosing period of 90 days and the postdosing recovery period of 28 days. Animals from different dose groups and reversal group showed normal body weight gain along with normal food consumption when compared with those of control animals. Toxic influence of any administered substance results in the altered physiological homeostasis that can be seen or observed through phenotypic changes or behaviours of an animal. To understand the nature of GutGard, the following phenotypic markers were assessed. The results of ophthalmic examination, detailed clinical observations including home cage observations, handling observations, and open field observations, and functional observations of treated animal groups confirmed normal physiological responses that were similar to results of control group animals.

Although statistically significant increase was observed in HCT, MCV, MCH, and platelets in male rats, the same trend was not found in female rats and moreover increased values were within normal range. Hence, these variations were considered as biologically insignificant and incidental. In the same manner, statistically significant increase was found in WBC count which was also within normal range. So, these variations in blood profile cannot be considered as affirmative pathological changes as no correlation was observed. Moreover, these variations were not considered as treatment related due to the lack of dose dependency. Hence, the observed alterations can be considered as incidental and clinically/biologically nonsignificant.

At the end of the dosing period on day 91, biochemical analyses showed statistically significant increase in the values of creatinine and chloride and statistically significant decrease in the value of potassium, total protein, calcium, albumin, phosphorus, sodium, and total cholesterol. All the observed changes were found to be within the normal limits and increased/decreased pattern was not similar in both sexes. Hence, statistically significant changes were considered as incidental and neither having any toxicological relevance nor clinical significance due to the lack of dose dependency. Apart from biochemical profile, although statistically significant changes were observed in the values of relative organ weights of liver, kidney, and adrenals, no related gross pathological and/or histopathological findings were seen and also dose dependency was not found; hence these findings were considered to be of no toxicological importance and moreover these types of biologically insignificant alterations occur commonly while performing toxicological studies using rodents [21–23].

Further, GutGard at 150 mg/day for 60 days in humans demonstrated the lack of treatment related adverse effects and corroborates the safety and substantiating its nontoxic nature [6, 7].

## 6. Conclusion

NOAEL (no observed adverse effect level) can be defined as the exposure level at which the frequency or severity of adverse effects between the exposed population and its appropriate control does not show statistical or biological increase; there can be some effects at this level, but they cannot be considered as adverse, nor precursors to specific adverse effects [24].

The median lethal dose of GutGard can be considered as >5000 mg/kg b.w, as it is devoid of any biologically relevant toxicity on single oral administration in female Sprague Dawley rats. It was also proved that it is free from marked signs of significant toxicity on continuous oral administration for 90 days at all the tested dose levels including the highest dose level of 1000 mg/kg body weight in both sexes of rats. Hence, the no observed adverse effect level (NOAEL) for GutGard in 90-day subchronic oral toxicity was found to be 1000 mg/kg b.w.

## Figures and Tables

**Table 1:** Effect of GutGard on body weight and gross pathology during 14-day (acute) oral toxicity.

Dose (mg/kg)	Mean body weight			Change in body weight (0–7) %	Change in body weight (7–14) %	Change in body weight (0–14) %	Gross pathological changes
	Day 0	Day 7	Day 14				
5000	201.73 ± 3.63	214.30 ± 3.51	226.40 ± 3.43	6.23 ± 0.22	5.65 ± 0.24	12.23 ± 0.32	Not observed

Mean ± SD (*n* = 3).

**Table 2:** Effect of GutGard on body weight of animals during 14D repeated toxicity study.

Group	Dose (mg/kg)	Day						
		0	1	4	8	11	14	15
*(a) Effect of GutGard on body weight of male rats during 14-day repeated toxicity study*								
I	0	141.06 ± 9.73	147.04 ± 10.81	166.22 ± 10.91	191.08 ± 19.18	209.50 ± 21.81	220.80 ± 25.64	203.38 ± 24.41
II	250	139.72 ± 10.43	144.96 ± 10.50	164.60 ± 8.05	184.74 ± 10.03	200.72 ± 11.15	206.54 ± 11.48	187.62 ± 13.90
III	500	139.68 ± 9.75	144.22 ± 9.38	161.42 ± 15.13	179.60 ± 27.18	195.90 ± 33.38	205.64 ± 31.65	186.10 ± 29.17
IV	1000	140.94 ± 7.76	144.88 ± 9.57	160.76 ± 12.13	178.86 ± 20.02	192.86 ± 26.75	205.46 ± 29.93	187.28 ± 27.66

*(b) Effect of GutGard on body weight of female rats during 14-day repeated toxicity study*								
I	0	130.18 ± 5.12	133.68 ± 5.98	142.64 ± 9.57	157.08 ± 11.47	167.46 ± 14.70	173.44 ± 16.94	156.24 ± 13.86
II	250	130.14 ± 5.17	134.10 ± 4.38	145.42 ± 6.04	159.28 ± 9.44	167.84 ± 12.13	172.66 ± 11.14	157.78 ± 8.89
III	500	130.52 ± 4.70	134.56 ± 5.74	141.28 ± 9.09	150.52 ± 12.44	157.60 ± 12.05	163.42 ± 12.36	148.94 ± 11.23
IV	1000	130.96 ± 4.35	134.18 ± 6.00	140.24 ± 6.72	153.30 ± 7.74	162.64 ± 8.17	166.34 ± 7.60	151.02 ± 6.28

Mean ± SD (*n* = 5).

**Table 3:** Effect of GutGard on haematological parameters of male rats during 90 D oral toxicity study.

Parameter	Dose (mg/kg)					
	0	0 (Rev)^$^	250	500	1000	1000 (Rev)^$^
Hb (g/dL)	14.99 ± 0.61	16.26 ± 0.62	15.53 ± 1.29	15.75 ± 0.89	14.79 ± 0.70	16.64 ± 0.66
RBC × 10^6^ (/*μ*L)	8.54 ± 0.51	9.67 ± 0.34	8.59 ± 0.87	9.12 ± 0.63	7.97 ± 0.37	9.51 ± 0.69
HCT (%)	41.99 ± 2.03	47.62 ± 1.61	43.42 ± 3.66	45.29 ± 3.18^*∗*^	41.06 ± 1.86	47.38 ± 2.98
MCV (fL)	49.22 ± 1.36	49.26 ± 1.76	50.68 ± 1.98	49.67 ± 1.82	51.55 ± 1.50^*#*^	49.86 ± 1.86
MCH (pg)	17.59 ± 0.55	16.82 ± 0.56	18.16 ± 0.75	17.31 ± 1.24	18.57 ± 0.60^*∗*^	17.56 ± 0.69
MCHC (g/dL)	35.73 ± 0.44	34.10 ± 0.64	35.84 ± 0.72	34.86 ± 2.00	36.01 ± 0.54	35.22 ± 1.24
PLTS × 10^3^(/*μ*L)	319.50 ± 118.44	440.20 ± 73.57	411.50 ± 61.47	416.10 ± 90.78	423.20 ± 80.72^*∗*^	366.40 ± 33.50
WBC × 10^3^ (/*μ*L)	8.57 ± 3.35	9.50 ± 1.78	13.65 ± 4.48^*∗*^	12.08 ± 4.16	13.31 ± 5.17^*∗*^	9.56 ± 2.82
Neutrophils (%)	21.40 ± 4.27	21.20 ± 3.70	20.40 ± 4.12	21.90 ± 3.70	20.30 ± 4.45	21.40 ± 4.51
Lymphocytes (%)	74.90 ± 3.84	75.00 ± 3.39	76.50 ± 4.14	75.20 ± 3.39	76.10 ± 4.98	75.40 ± 4.04
Eosinophils (%)	1.30 ± 0.67	1.20 ± 0.84	0.70 ± 0.82	0.80 ± 0.92	1.20 ± 0.92	1.00 ± 1.00
Monocytes (%)	2.40 ± 0.52	2.60 ± 0.55	2.40 ± 0.70	2.10 ± 0.57	2.40 ± 0.70	2.20 ± 0.84
Basophils (%)	0.00 ± 0.00	0.00 ± 0.00	0.00 ± 0.00	0.00 ± 0.00	0.00 ± 0.00	0.00 ± 0.00
PT (sec)	14.90 ± 3.21	14.80 ± 3.56	14.60 ± 3.10	14.70 ± 2.75	15.10 ± 3.28	15.40 ± 3.44

Mean ± SD (*n* = 10); ^$^*n* = 5. Hb: hemoglobin; RBC: red blood corpuscles; HCT: hematocrit; MCV: mean corpuscular volume; MCH: mean corpuscular hemoglobin; MCHC: mean corpuscular hemoglobin concentration; WBC: white blood corpuscles; N: neutrophils; L: lymphocytes; E: eosinophils; M: monocytes; B: basophils; PT: prothrombin time; PLTS: platelets; Rev: reversal. ^*∗*^ = significant at 95% level of confidence (*p* ≤ 0.05); ^*#*^ = significant at 99% level of confidence (*p* ≤ 0.01).

**Table 4:** Effect of GutGard on haematological parameters of female rats during 90 D oral toxicity study.

Parameter	Dose (mg/kg)					
	0	0 (Rev)^$^	250	500	1000	1000 (Rev)^$^
Hb (g/dL)	16.29 ± 1.56	15.64 ± 0.29	16.10 ± 2.08	15.78 ± 2.08	15.34 ± 0.75	16.02 ± 1.51
RBC × 10^6^ (/*μ*L)	8.56 ± 0.45	8.47 ± 0.40	8.54 ± 0.95	8.30 ± 1.05	8.12 ± 0.59	8.46 ± 0.95
HCT (%)	44.45 ± 3.92	43.70 ± 1.57	44.17 ± 5.92	42.89 ± 4.99	42.01 ± 3.18	43.98 ± 4.45
MCV (fL)	51.89 ± 2.24	51.62 ± 0.73	51.63 ± 1.50	51.71 ± 1.94	51.76 ± 1.82	52.04 ± 1.17
MCH (pg)	19.00 ± 1.17	18.48 ± 0.58	18.84 ± 0.66	19.03 ± 0.90	18.91 ± 0.81	18.98 ± 0.56
MCHC (g/dL)	36.64 ± 1.12	35.80 ± 0.77	36.46 ± 0.60	36.78 ± 0.77	36.58 ± 1.44	36.48 ± 0.37
PLTS × 10^3^(/*μ*L)	336.60 ± 88.50	360.20 ± 70.46	363.80 ± 93.03	392.90 ± 72.10	422.80 ± 55.47	335.80 ± 79.33
WBC × 10^3^ (/*μ*L)	9.74 ± 3.85	7.32 ± 5.28	7.97 ± 2.88	9.73 ± 3.93	15.73 ± 5.02^*#*^	4.38 ± 0.86
Neutrophils (%)	22.50 ± 4.30	20.80 ± 3.27	21.20 ± 4.37	20.90 ± 4.15	21.40 ± 5.30	21.20 ± 4.21
Lymphocytes (%)	74.20 ± 3.58	75.80 ± 2.59	75.80 ± 4.21	76.10 ± 3.51	75.80 ± 5.03	75.80 ± 3.90
Eosinophils (%)	0.90 ± 0.74	1.00 ± 0.71	0.70 ± 0.67	0.90 ± 0.88	0.60 ± 0.70	0.80 ± 0.84
Monocytes (%)	2.40 ± 0.70	2.40 ± 0.55	2.30 ± 0.48	2.10 ± 0.57	2.20 ± 0.63	2.20 ± 0.84
Basophils (%)	0.00 ± 0.00	0.00 ± 0.00	0.00 ± 0.00	0.00 ± 0.00	0.00 ± 0.00	0.00 ± 0.00
PT (sec)	15.10 ± 3.07	15.40 ± 3.85	15.60 ± 3.44	15.40 ± 3.06	15.60 ± 2.95	14.80 ± 4.32

Mean ± SD (*n* = 10); ^$^*n* = 5. Hb: hemoglobin; RBC: red blood corpuscles; HCT: hematocrit; MCV: mean corpuscular volume; MCH: mean corpuscular hemoglobin; MCHC: mean corpuscular hemoglobin concentration; WBC: white blood corpuscles; N: neutrophils; L: lymphocytes; E: eosinophils; M: monocytes; B: basophils; PT: prothrombin time; PLTS: platelets; Rev: reversal. ^*∗*^  = significant at 95% level of confidence (*p* ≤ 0.05); ^*#*^  = significant at 99% level of confidence (*p* ≤ 0.01).

**Table 5:** Effect of GutGard on biochemistry parameters of male rats during 90 D oral toxicity study.

Parameter	Dose (mg/kg)					
	0	0 (Rev)^$^	250	500	1000	1000 (Rev)^$^
TP (g/dL)	6.90 ± 0.56	6.69 ± 0.44	6.82 ± 0.49	6.93 ± 0.63	6.81 ± 0.46	6.18 ± 0.72
BUN (mg/dL)	15.30 ± 2.31	13.40 ± 1.14	14.80 ± 2.44	16.10 ± 1.45	15.90 ± 2.13	14.00 ± 0.71
Urea (mg/dL)	33.35 ± 5.04	29.21 ± 2.49	32.26 ± 5.32	35.10 ± 3.16	34.66 ± 4.65	30.52 ± 1.54
ALT (U/L)	63.50 ± 12.72	52.80 ± 8.67	58.60 ± 11.15	62.10 ± 8.52	62.30 ± 9.58	42.40 ± 9.71
AST (U/L)	112.70 ± 25.18	98.40 ± 27.42	107.80 ± 17.01	110.70 ± 21.28	97.10 ± 9.87	101.40 ± 37.92
ALP (U/L)	193.50 ± 18.36	126.80 ± 33.76	198.80 ± 31.57	193.60 ± 23.44	182.20 ± 32.76	90.20 ± 38.49
Glucose (mg/dL)	84.50 ± 7.41	82.80 ± 13.03	87.90 ± 13.96	83.00 ± 11.84	86.40 ± 6.08	86.20 ± 10.50
Cal (mmol/L)	3.54 ± 0.53	3.69 ± 0.18	3.55 ± 0.27	3.31 ± 0.22	3.64 ± 0.18	3.44 ± 0.16^*∗*^
P (mg/dL)	7.20 ± 0.72	5.76 ± 1.03	7.37 ± 0.75	7.73 ± 0.86	7.05 ± 0.55	5.10 ± 0.62
GGTP (U/L)	10.53 ± 2.10	7.59 ± 2.13	9.53 ± 3.01	10.80 ± 1.90	8.69 ± 1.17	7.90 ± 1.54
TB (mg/dL)	0.29 ± 0.09	0.21 ± 0.07	0.25 ± 0.07	0.26 ± 0.06	0.21 ± 0.05	0.24 ± 0.04
Albumin (g/dL)	2.01 ± 0.13	2.08 ± 0.30	2.09 ± 0.15	2.05 ± 0.19	2.09 ± 0.17	1.84 ± 0.29
Creatinine (mg/dL)	0.53 ± 0.31	0.98 ± 0.10	1.13 ± 0.52^*#*^	1.01 ± 0.29^*∗*^	0.70 ± 0.23	0.89 ± 0.16
Sodium (mmol/L)	149.27 ± 1.74	156.44 ± 0.98	151.02 ± 1.90	148.88 ± 2.75	147.45 ± 1.46	147.50 ± 1.87^*#*^
Potassium (mmol/L)	4.65 ± 0.36	4.02 ± 0.17	4.05 ± 0.41^*#*^	4.50 ± 0.40	4.47 ± 0.33	4.04 ± 0.48
Chloride (mmol/L)	94.70 ± 2.90	92.25 ± 1.65	95.37 ± 4.02	98.84 ± 4.46^*#*^	89.51 ± 2.13	98.59 ± 4.95
TC (mmol/L)	34.02 ± 7.58	39.55 ± 13.42	32.18 ± 5.43	36.60 ± 5.97	30.80 ± 5.34	29.89 ± 9.13
TG (mmol/L)	42.40 ± 18.11	41.00 ± 10.07	34.80 ± 8.39	38.90 ± 9.16	41.00 ± 12.10	36.80 ± 11.17

Mean ± SD (*n* = 10); ^$^*n* = 5. TP: total protein; BUN: blood urea nitrogen; ALT: alanine aminotransferase; AST: aspartate aminotransferase; ALP: alkaline phosphatase; Cal: calcium; P: phosphorus; TB: total bilirubin; GGTP: gamma glutamyl transferase; TG: triglycerides; TC: total cholesterol; Rev.: reversal. ^*∗*^= significant at 95% level of confidence (*p* ≤ 0.05); ^*#*^  = significant at 99% level of confidence (*p* ≤ 0.01).

**Table 6:** Effect of GutGard on biochemistry parameters of female rats during 90 D oral toxicity study.

Parameter	Dose (mg/kg)					
	0	0 (Rev)^$^	250	500	1000	1000 (Rev)^$^
TP (g/dL)	7.26 ± 0.55	7.33 ± 0.30	6.74 ± 0.44	6.58 ± 0.63^*∗*^	7.37 ± 0.60	7.29 ± 0.77
BUN (mg/dL)	18.50 ± 2.07	13.80 ± 1.10	17.60 ± 2.95	19.20 ± 2.94	18.80 ± 4.47	14.80 ± 1.30
Urea (mg/dL)	40.33 ± 4.51	30.08 ± 2.39	38.37 ± 6.43	41.86 ± 6.40	40.98 ± 9.74	32.26 ± 2.84
ALT (U/L)	51.00 ± 10.61	38.00 ± 1.58	48.90 ± 7.40	51.80 ± 4.78	62.90 ± 25.51	35.60 ± 3.71
AST (U/L)	104.00 ± 11.55	82.80 ± 4.87	89.70 ± 15.92	93.50 ± 11.93	100.50 ± 21.59	78.80 ± 5.93
ALP (U/L)	88.10 ± 19.50	58.20 ± 6.76	86.10 ± 9.23	91.60 ± 23.63	110.80 ± 32.70	63.60 ± 9.86
Glucose (mg/dL)	74.00 ± 14.93	68.80 ± 15.16	67.50 ± 16.72	77.60 ± 9.58	88.90 ± 20.08	86.40 ± 18.24
Calcium (mmol/L)	3.52 ± 0.12	3.79 ± 0.08	3.44 ± 0.40	3.08 ± 0.30^*#*^	3.53 ± 0.11	3.75 ± 0.06
Phosphorus (mg/dL)	7.23 ± 1.06	4.54 ± 0.62	5.68 ± 0.74^*#*^	6.13 ± 0.63^*∗*^	5.80 ± 1.00^*#*^	4.22 ± 0.36
GGTP (U/L)	7.38 ± 2.74	9.68 ± 2.28	8.63± 2.77	6.30 ± 1.90	8.91 ± 2.34	7.91 ± 2.28
TB (mg/dL)	0.48 ± 0.12	0.31 ± 0.06	0.41 ± 0.10	0.49 ± 0.21	0.47 ± 0.10	0.37 ± 0.05
Albumin (g/dL)	2.26 ± 0.21	2.27 ± 0.11	2.20 ± 0.28	1.99 ± 0.22^*∗*^	2.34 ± 0.24	2.31 ± 0.22
Creatinine (mg/dL)	1.18 ± 0.25	0.96 ± 0.06	0.88 ± 0.16	1.31 ± 0.41	1.39 ± 0.34	1.04 ± 0.16
Sodium (mmol/L)	150.04 ± 1.59	146.31 ± 0.73	151.28 ± 2.96	148.74 ± 2.36	148.49 ± 1.10^*∗*^	145.04 ± 1.46
Potassium (mmol/L)	4.24 ± 0.25	3.91 ± 0.08	3.92 ± 0.58	4.33 ± 0.47	4.16 ± 0.22	4.22 ± 0.44
Chloride (mmol/L)	97.10 ± 1.56	97.92 ± 2.55	93.22 ± 6.37	98.37 ± 2.14	95.79 ± 2.82	94.91 ± 2.96
TC (mg/dL)	39.44 ± 6.68	41.99 ± 2.94	32.87 ± 6.66^*∗*^	39.61 ± 18.86	42.20 ± 6.03	43.54 ± 5.01
TGL (mg/dL)	53.90 ± 7.89	61.40 ± 22.03	50.10 ± 10.27	48.60 ± 8.76	56.70 ± 13.90	56.80 ± 16.41

Mean ± SD (*n* = 10); ^$^*n* = 5. TP: total protein; BUN: blood urea nitrogen; ALT: alanine aminotransferase; AST: aspartate aminotransferase; ALP: alkaline phosphatase; Cal: calcium; P: phosphorus; TB: total bilirubin; GGTP: gamma glutamyl transferase; TG: triglycerides; TC: total cholesterol; Rev.: reversal. ^*∗*^  = significant at 95% level of confidence (*p* ≤ 0.05); ^*#*^  = significant at 99% level of confidence (*p* ≤ 0.01).

**Table 7:** Effect of GutGard on urine parameters of male rats during 90 D oral toxicity study.

Parameter	Dose (mg/kg)					
	0	0 (Rev)^$^	250	500	1000	1000 (Rev)^$^
Volume (ml)	4.81 ± 0.76	5.10 ± 0.20	5.07 ± 0.74	5.23 ± 0.47	5.00 ± 0.58	5.20 ± 0.45
Glucose (mmol/L)	0.00 ± 0.00	0.00 ± 0.00	0.00 ± 0.00	0.00 ± 0.00	0.00 ± 0.00	0.00 ± 0.00
Bilirubin (mmol/L)	0.00 ± 0.00	0.00 ± 0.00	0.00 ± 0.00	0.00 ± 0.00	0.00 ± 0.00	0.00 ± 0.00
Ketones (mmol/L)	0.00 ± 0.00	0.00 ± 0.00	0.00 ± 0.00	0.00 ± 0.00	0.00 ± 0.00	0.00 ± 0.00
Sp.Gr.(g/L)	1.028 ± 0.003	1.027 ± 0.003	1.028 ± 0.003	1.028 ± 0.003	1.028 ± 0.003	1.028 ± 0.003
OB (ca CELL/*μ*L)	0.00 ± 0.00	0.00 ± 0.00	0.00 ± 0.00	0.00 ± 0.00	0.00 ± 0.00	0.00 ± 0.00
pH	6.20 ± 0.26	6.30 ± 0.27	6.25 ± 0.26	6.25 ± 0.26	6.25 ± 0.26	6.20 ± 0.27
URB (mmol/L)	0.00 ± 0.00	0.00 ± 0.00	0.00 ± 0.00	0.00 ± 0.00	0.00 ± 0.00	0.00 ± 0.00
Nitrite	0.00 ± 0.00	0.00 ± 0.00	0.00 ± 0.00	0.00 ± 0.00	0.00 ± 0.00	0.00 ± 0.00

Mean ± SD (*n* = 10); $ *n* = 5. Rev.: reversal; OB: occult blood; URB: urobilinogen; Sp.Gr.: specific gravity.

**Table 8:** Effect of GutGard on urine parameters of female rats during 90 D oral toxicity study.

Parameter	Dose (mg/kg)					
	0	0 (Rev)^$^	250	500	1000	1000 (Rev)^$^
Volume (ml)	4.71 ± 0.38	5.06 ± 0.65	5.13 ± 0.27	5.21 ± 0.31	4.89 ± 0.48	4.96 ± 0.82
Glucose (mmol/L)	0.00 ± 0.00	0.00 ± 0.00	0.00 ± 0.00	0.00 ± 0.00	0.00 ± 0.00	0.00 ± 0.00
Bilirubin (mmol/L)	0.00 ± 0.00	0.00 ± 0.00	0.00 ± 0.00	0.00 ± 0.00	0.00 ± 0.00	0.00 ± 0.00
Ketones (mmol/L)	0.00 ± 0.00	0.00 ± 0.00	0.00 ± 0.00	0.00 ± 0.00	0.00 ± 0.00	0.00 ± 0.00
Sp.Gr. (g/L)	1.028 ± 0.003	1.028 ± 0.003	1.028 ± 0.003	1.029 ± 0.002	1.029 ± 0.002	1.027 ± 0.003
OB (ca CELL/*μ*L)	0.00 ± 0.00	0.00 ± 0.00	0.00 ± 0.00	0.00 ± 0.00	0.00 ± 0.00	0.00 ± 0.00
pH	6.25 ± 0.26	6.30 ± 0.27	6.20 ± 0.26	6.25 ± 0.26	6.25 ± 0.26	6.30 ± 0.27
URB (mmol/L)	0.00 ± 0.00	0.00 ± 0.00	0.00 ± 0.00	0.00 ± 0.00	0.00 ± 0.00	0.00 ± 0.00
Nitrite	0.00 ± 0.00	0.00 ± 0.00	0.00 ± 0.00	0.00 ± 0.00	0.00 ± 0.00	0.00 ± 0.00

Mean ± SD (*n* = 10); ^$^*n* = 5. Rev.: reversal; OB: occult blood; URB: urobilinogen; Sp.Gr.: specific gravity.

**Table 9:** Effect of GutGard on absolute organ weights (g) of male rats during 90 D oral toxicity study.

Parameter	Dose (mg/kg)					
	0	0 (Rev)^$^	250	500	1000	1000 (Rev)^$^
TBD (g)	372.56 ± 34.42	410.04 ± 25.95	359.73 ± 32.26	360.31 ± 32.48	350.15 ± 36.39	378.96 ± 14.02
Brain	1.943 ± 0.096	2.000 ± 0.137	1.943 ± 0.077	1.954 ± 0.080	1.949 ± 0.098	2.007 ± 0.109
Liver	11.258 ± 2.396	12.670 ± 2.026	10.931 ± 1.120	12.682 ± 2.422	13.189 ± 1.925	11.424 ± 0.824
Kidneys	2.622 ± 0.360	3.014 ± 0.304	2.596 ± 0.179	2.705 ± 0.358	2.889 ± 0.295	2.644 ± 0.219
Adrenals	0.0486 ± 0.0071	0.0472 ± 0.0061	0.0565 ± 0.0126	0.0533 ± 0.0052	0.0533 ± 0.0074	0.0432 ± 0.0071
Testes	2.851 ± 0.413	3.032 ± 0.082	2.937 ± 0.157	2.938 ± 0.290	2.860 ± 0.228	2.859 ± 0.192
Heart	1.197 ± 0.174	1.387 ± 0.184	1.178 ± 0.101	1.237 ± 0.212	1.166 ± 0.140	1.339 ± 0.155
Spleen	1.379 ± 0.482	1.148 ± 0.224	1.049 ± 0.261	1.308 ± 0.289	1.379 ± 0.174	0.956 ± 0.115
Thymus	0.210 ± 0.068	0.175 ± 0.059	0.160 ± 0.060	0.200 ± 0.053	0.147 ± 0.040	0.147 ± 0.022
Epididymis	1.182 ± 0.086	1.201 ± 0.151	1.068 ± 0.081	1.099 ± 0.134	1.130 ± 0.088	1.074 ± 0.061

Mean ± SD (*n* = 10); ^$^*n* = 5; Rev.: reversal; TBD: total body weight.

**Table 10:** Effect of GutGard on relative organ weights (%) of male rats during 90 D oral toxicity study.

Parameter	Dose (mg/kg)					
	0	0 (Rev)^$^	250	500	1000	1000 (Rev)^$^
Brain	0.525 ± 0.052	0.490 ± 0.054	0.544 ± 0.056	0.545 ± 0.033	0.562 ± 0.067	0.531 ± 0.041
Liver	3.026 ± 0.639	3.083 ± 0.387	3.059 ± 0.415	3.497 ± 0.409	3.762 ± 0.361^*#*^	3.013 ± 0.155
Kidneys	0.705 ± 0.082	0.734 ± 0.046	0.726 ± 0.069	0.749 ± 0.054	0.827 ± 0.051^*#*^	0.697 ± 0.045
Adrenals	0.0131 ± 0.0018	0.0115 ± 0.0014	0.0159 ± 0.0040	0.0148 ± 0.0014^*∗*^	0.0153 ± 0.0022^*∗*^	0.0114 ± 0.0020
Testes	0.766 ± 0.095	0.743 ± 0.065	0.823 ± 0.087	0.818 ± 0.079	0.825 ± 0.106	0.755 ± 0.059
Heart	0.322 ± 0.038	0.339 ± 0.046	0.330 ± 0.045	0.344 ± 0.061	0.333 ± 0.024	0.353 ± 0.033
Spleen	0.371 ± 0.133	0.281 ± 0.058	0.297 ± 0.095	0.364 ± 0.074	0.399 ± 0.073	0.253 ± 0.036
Thymus	0.056 ± 0.018	0.043 ± 0.015	0.044 ± 0.017	0.056 ± 0.016	0.042 ± 0.010	0.039 ± 0.007
Epididymis	0.320 ± 0.037	0.294 ± 0.040	0.300 ± 0.041	0.305 ± 0.023	0.326 ± 0.039	0.284 ± 0.019

Mean ± SD (*n* = 10); ^$^*n* = 5; Rev.: reversal. ^*∗*^  = significant at 95% level of confidence (*p* ≤ 0.05); ^*#*^  = significant at 99% level of confidence (*p* ≤ 0.01).

**Table 11:** Effect of GutGard on absolute organ weights (g) of female rats during 90 D oral toxicity study.

Parameter	Dose (mg/kg)					
	0	0 (Rev)^$^	250	500	1000	1000 (Rev)^$^
TBD (g)	246.48 ± 19.99	260.50 ± 8.18	239.39 ± 18.31	230.75 ± 14.55	239.63 ± 18.72	249.70 ± 27.05
Brain	1.776 ± 0.160	1.851 ± 0.161	1.846 ± 0.107	1.845 ± 0.067	1.858 ± 0.061	1.804 ± 0.104
Liver	6.904 ± 0.738	7.848 ± 0.708	6.926 ± 0.492	7.532 ± 1.042	8.896 ± 1.333	7.137 ± 0.491
Kidneys	1.645 ± 0.133	1.692 ± 0.164	1.599 ± 0.122	1.729 ± 0.127	1.685 ± 0.081	1.542 ± 0.100
Adrenals	0.0641 ± 0.0151	0.0605 ± 0.0193	0.0568 ± 0.0067	0.0665 ± 0.0100	0.0642 ± 0.0156	0.0582 ± 0.0055
Ovaries	0.0838 ± 0.0233	0.1216 ± 0.0212	0.0787 ± 0.0150	0.0982 ± 0.0161	0.0920 ± 0.0221	0.0977 ± 0.0166
Heart	0.794 ± 0.081	1.002 ± 0.063	0.858 ± 0.061	0.842 ± 0.115	0.863 ± 0.074	0.809 ± 0.128
Spleen	0.870 ± 0.169	0.966 ± 0.157	0.772 ± 0.150	0.891 ± 0.199	0.922 ± 0.173	0.741 ± 0.039
Thymus	0.150 ± 0.051	0.188 ± 0.050	0.170 ± 0.040	0.187 ± 0.052	0.145 ± 0.062	0.146 ± 0.061
Uterus	0.358 ± 0.138	0.483 ± 0.091	0.377 ± 0.086	0.374 ± 0.114	0.336 ± 0.049	0.450 ± 0.100

Mean ± SD (*n* = 10); ^$^*n* = 5; Rev.: reversal; TBD: total body weight.

**Table 12:** Effect of GutGard on relative organ weights (%) of female rats during 90 D oral toxicity study.

Parameter	Dose (mg/kg)					
	0	0 (Rev)^$^	250	500	1000	1000 (Rev)^$^
Brain	0.723 ± 0.077	0.711 ± 0.056	0.774 ± 0.057	0.802 ± 0.051	0.780 ± 0.074	0.728 ± 0.080
Liver	2.812 ± 0.324	3.013 ± 0.254	2.904 ± 0.260	3.261 ± 0.372^*∗*^	3.718 ± 0.539^*#*^	2.891 ± 0.405
Kidneys	0.670 ± 0.070	0.649 ± 0.058	0.669 ± 0.048	0.750 ± 0.044^*#*^	0.750 ± 0.028	0.623 ± 0.076
Adrenals	0.0263 ± 0.0069	0.0234 ± 0.0083	0.0239 ± 0.0040	0.0289 ± 0.0041	0.0266 ± 0.0054	0.0236 ± 0.0041
Ovaries	0.0344 ± 0.0110	0.0467 ± 0.0082	0.0330 ± 0.0065	0.0429 ± 0.0082	0.0385 ± 0.0092	0.0395 ± 0.0078
Heart	0.323 ± 0.034	0.385 ± 0.026	0.360 ± 0.033^*∗*^	0.364 ± 0.039^*∗*^	0.360 ± 0.020^*∗*^	0.328 ± 0.065
Spleen	0.353 ± 0.062	0.371 ± 0.061	0.325 ± 0.073	0.385 ± 0.075	0.387 ± 0.079	0.299 ± 0.029^*∗*^
Thymus	0.060 ± 0.017	0.072 ± 0.019	0.071 ± 0.015	0.082 ± 0.025	0.060 ± 0.022	0.059 ± 0.025
Uterus	0.146 ± 0.058	0.186 ± 0.40	0.157 ± 0.033	0.163 ± 0.49	0.141 ± 0.018	0.184 ± 0.051

Mean ± SD (*n* = 10); ^$^*n* = 5; Rev.: reversal. ^*∗*^  = significant at 95% level of confidence (*p* ≤ 0.05); ^*#*^  = significant at 99% level of confidence (*p* ≤ 0.01).

## Data Availability

The data used to support the findings of this study are included within the article.

## Supplementary Materials

Supplementary Materials All the histopathology pictures were incorporated in Supplementary File.
