# Supplementary material for: Acute and Subchronic Toxicity Study of Flavonoid Rich Extract of Glycyrrhiza glabra (GutGard®) in Sprague Dawley Rats
Source: J Toxicol. 2022 Mar 31;2022:8517603. doi: 10.1155/2022/8517603 (PMC8989621; doi:10.1155/2022/8517603)
Supplement: Supplementary Materials — All the histopathology pictures were incorporated in Supplementary File. [file 8517603.f1.docx]

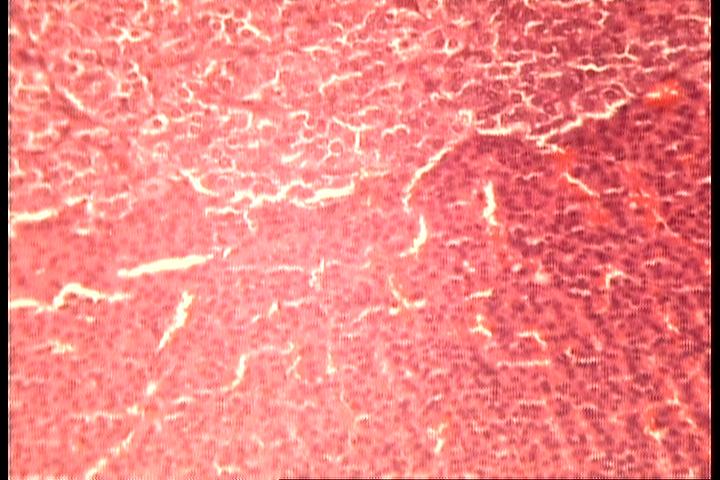

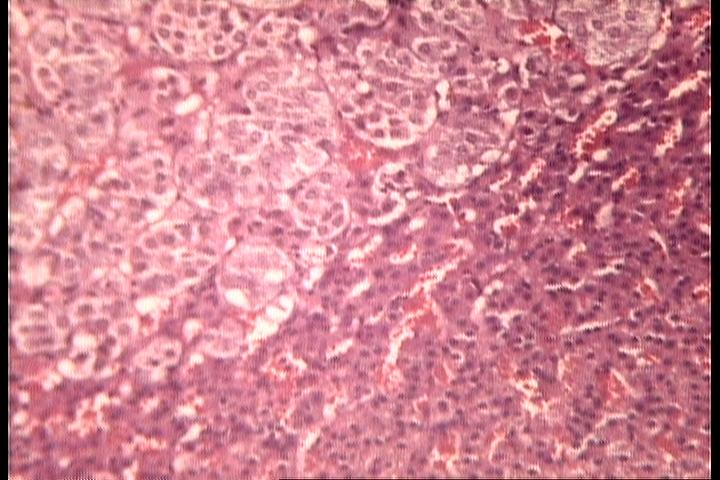

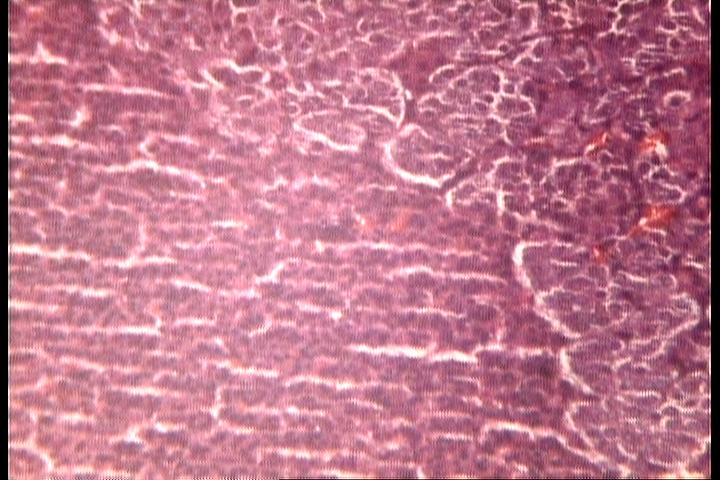

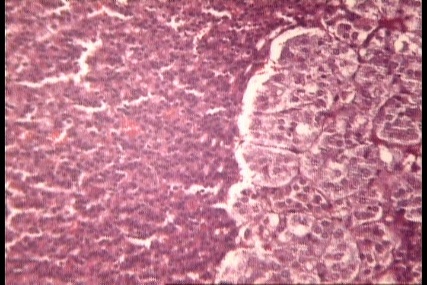
Adrenal gland

High dose - male

High dose - female

Ctrl - male

Ctrl - female


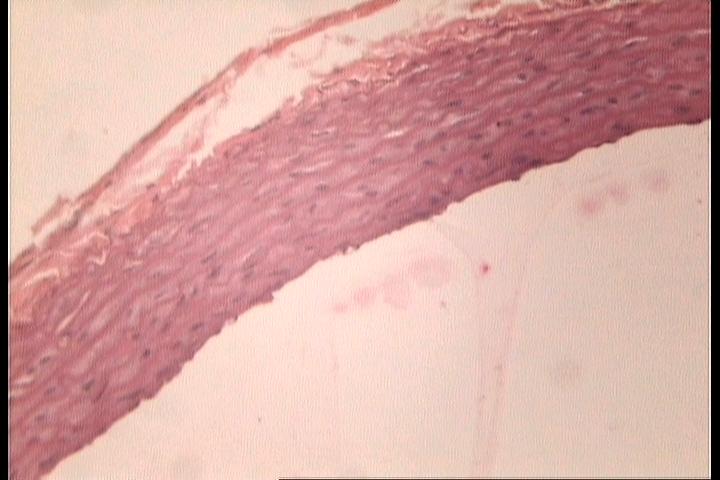

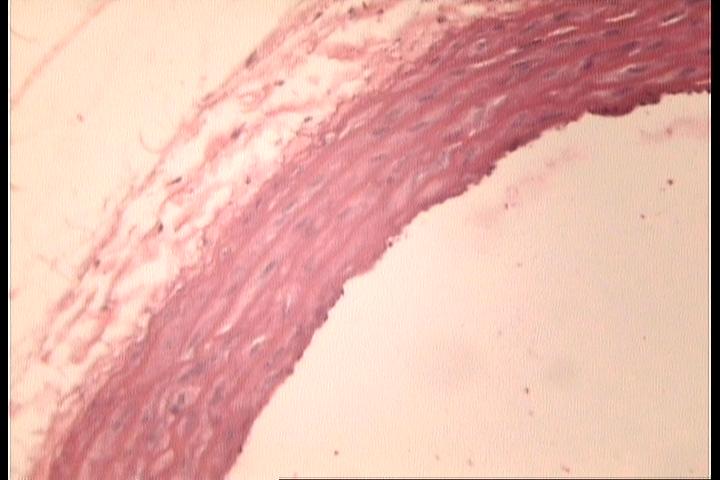

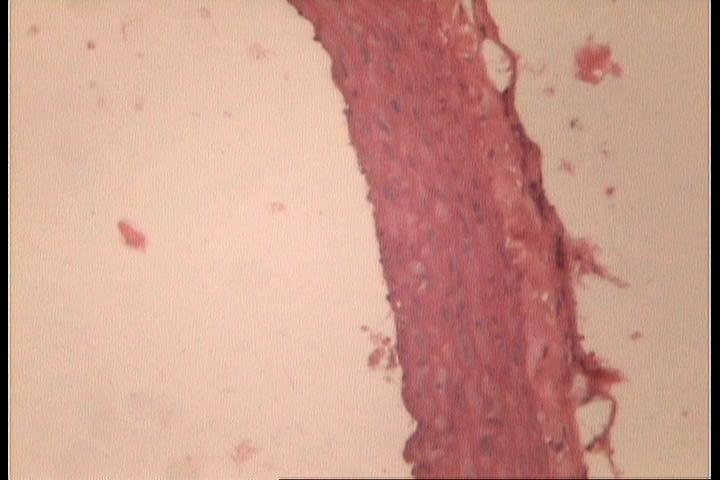

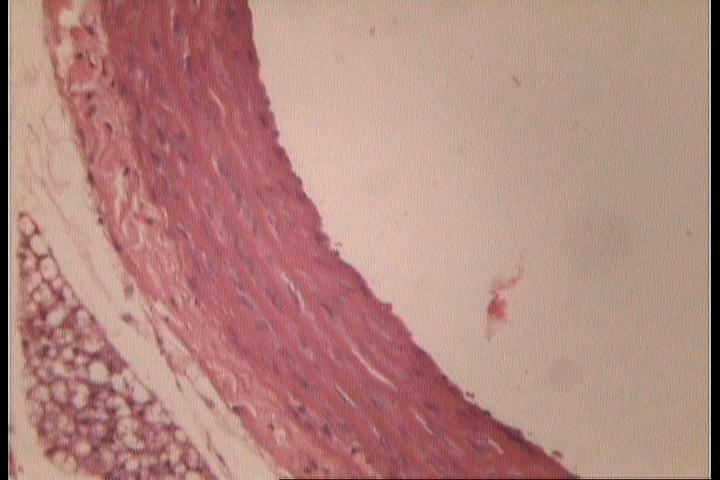
Aorta

High dose - male

High dose - female

Ctrl - female

Ctrl - male


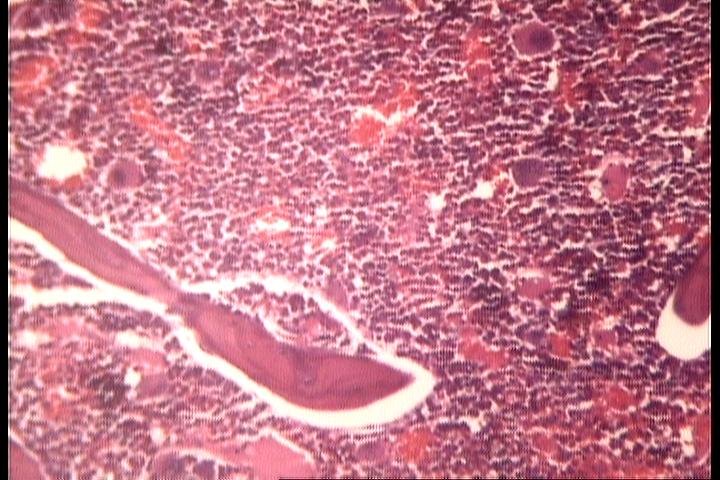

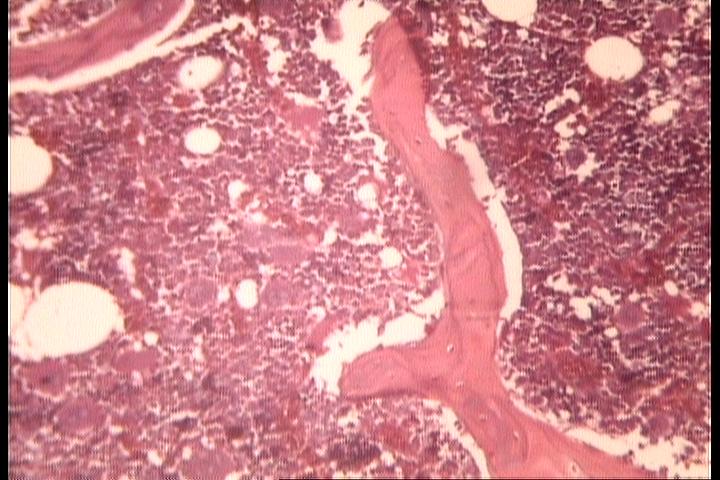

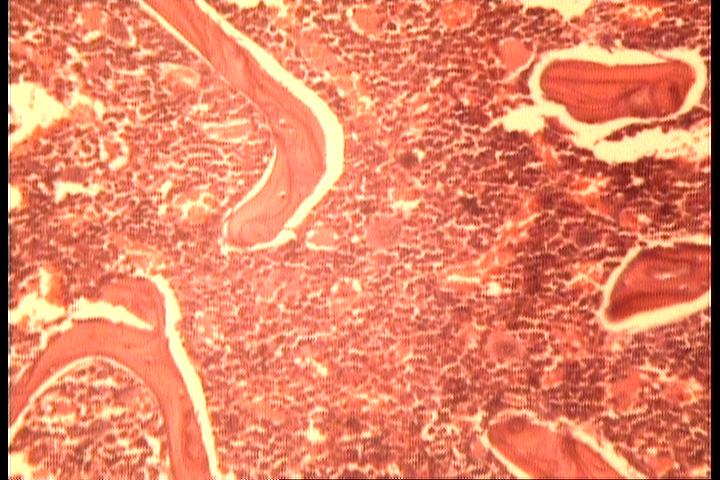

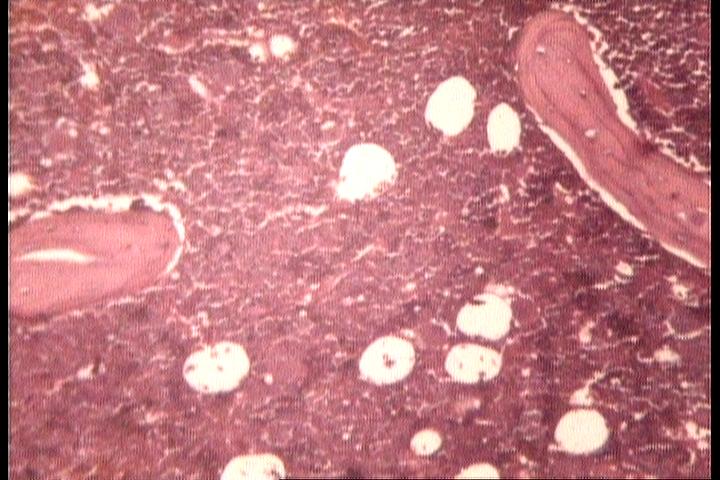
Bone marrow

High dose - male

High dose - female

Ctrl - male

Ctrl - female


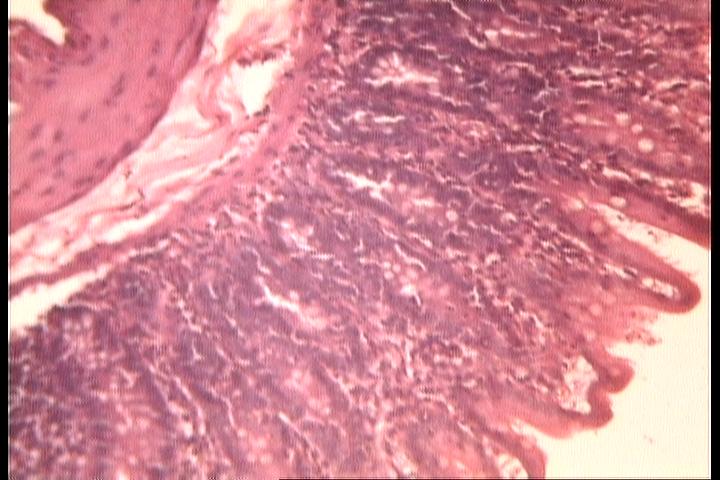

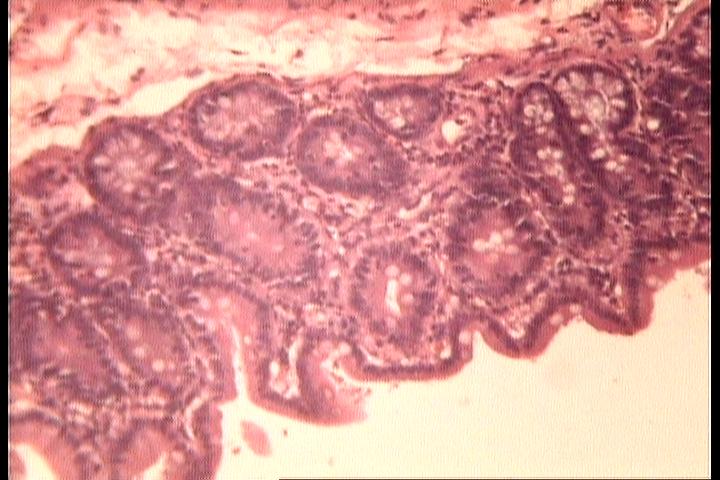

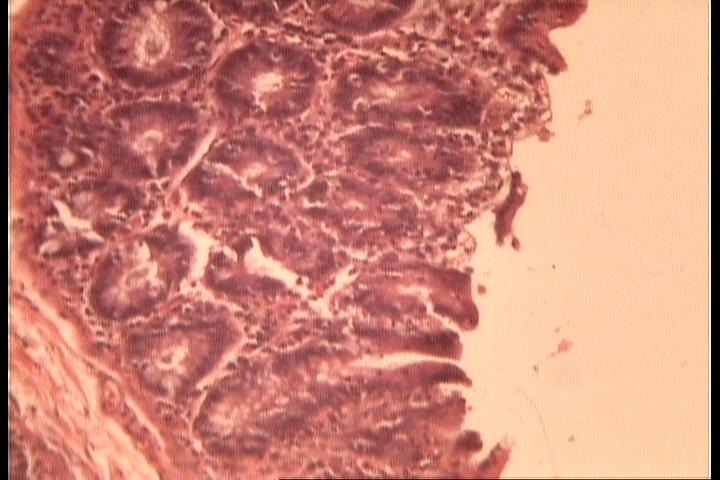

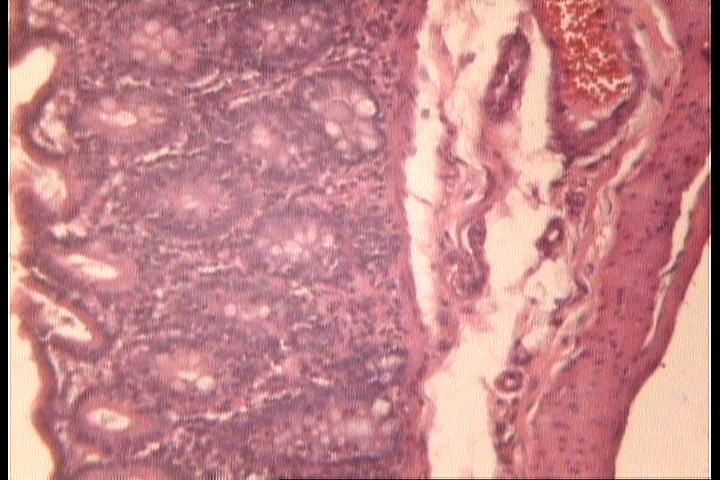
Caecum

High dose - male

High dose - female

Ctrl - male

Ctrl - female


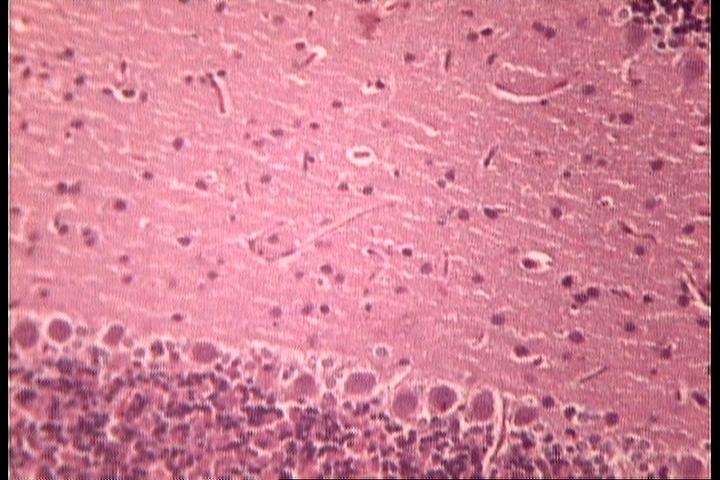

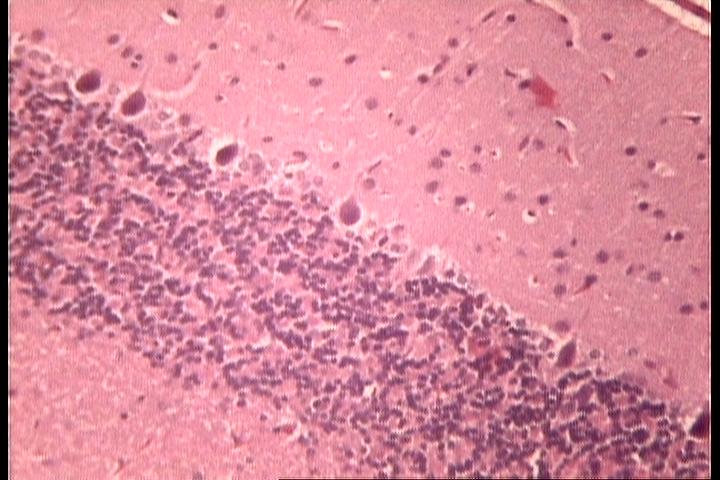

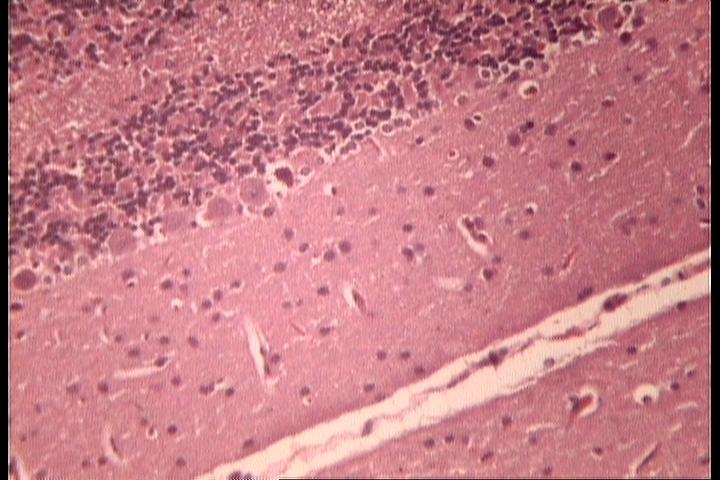

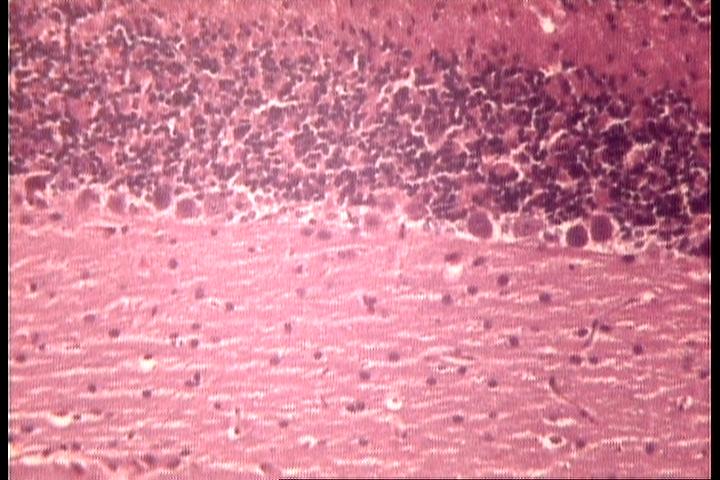
Cerebellum

High dose - male

High dose - female

Ctrl - male

Ctrl - female


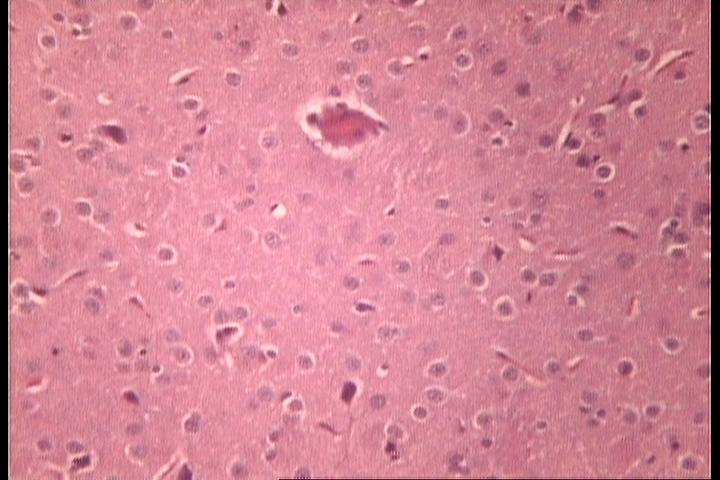

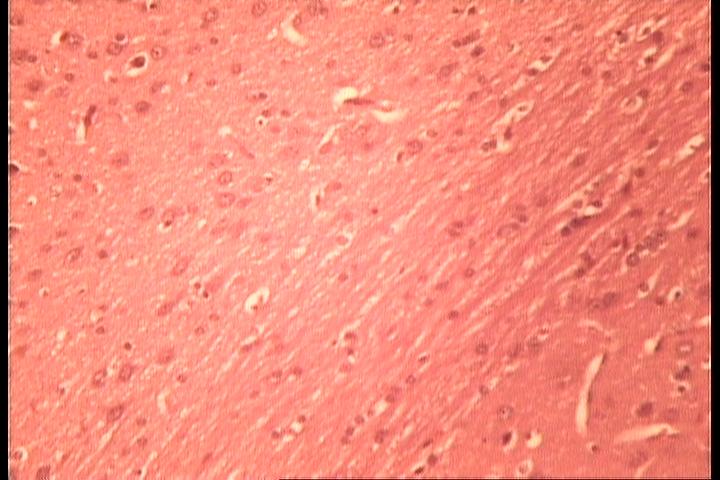

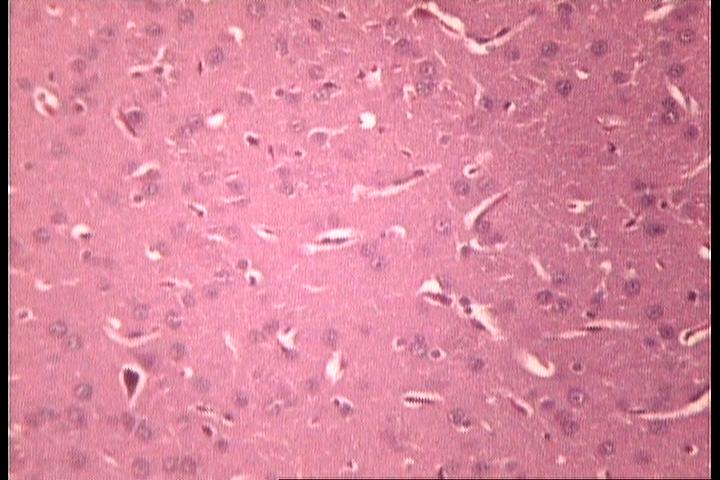

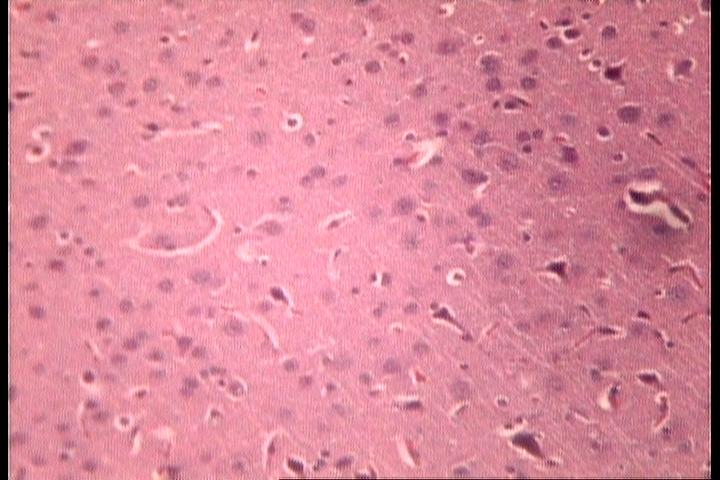
Cerebrum

High dose - male

High dose - female

Ctrl - male

Ctrl - female


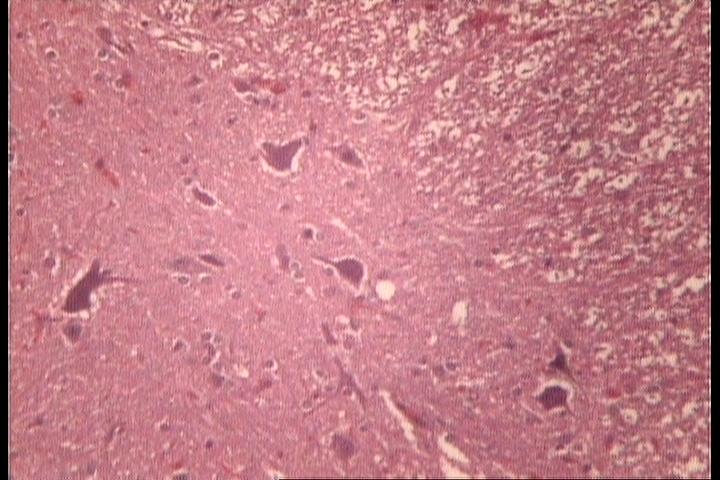

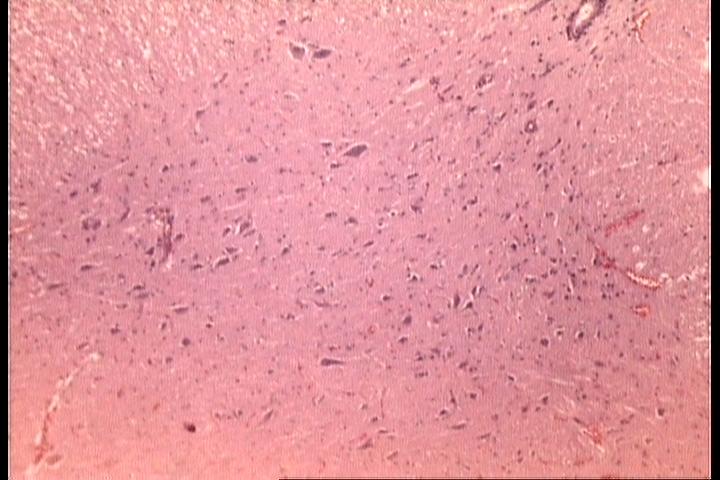

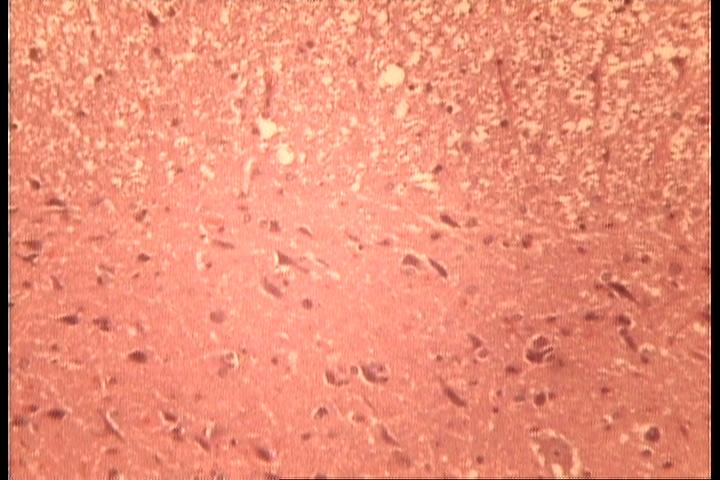

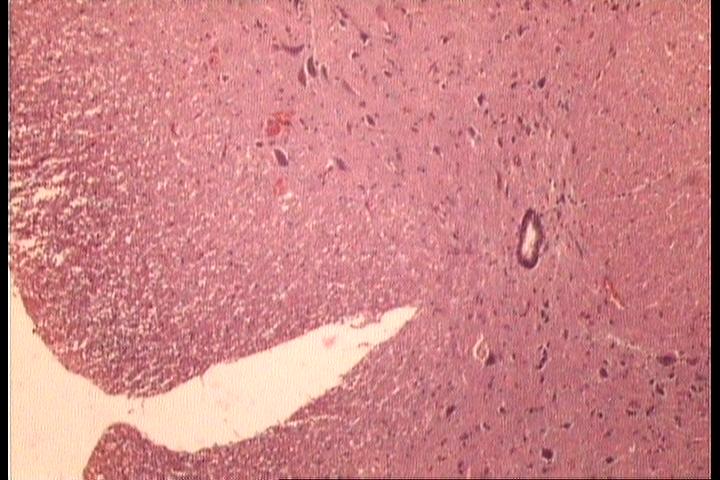
Cervical Spinal cord

High dose - male

High dose - female

Ctrl - male

Ctrl - female


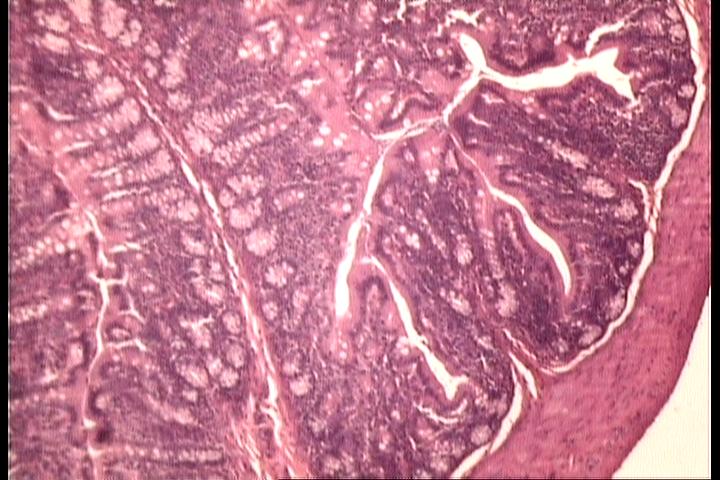

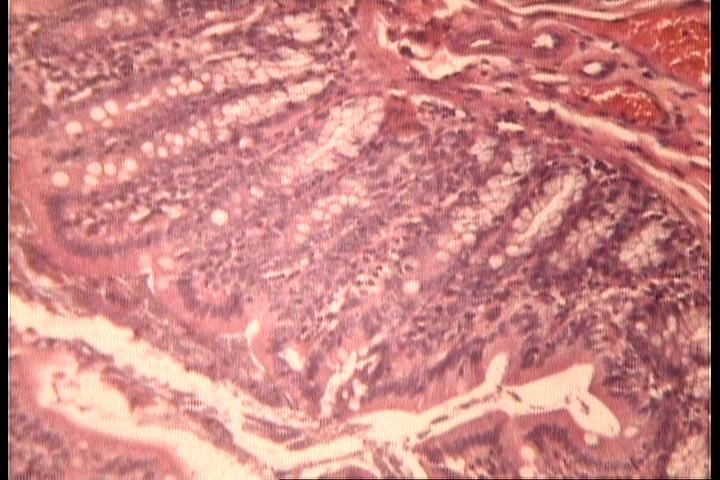

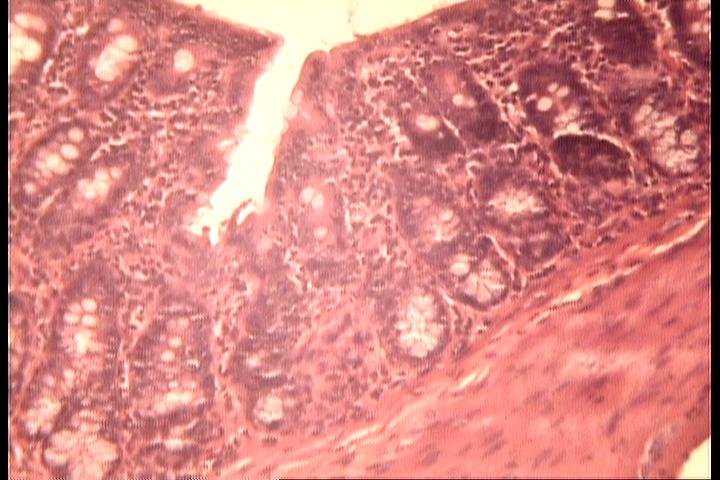

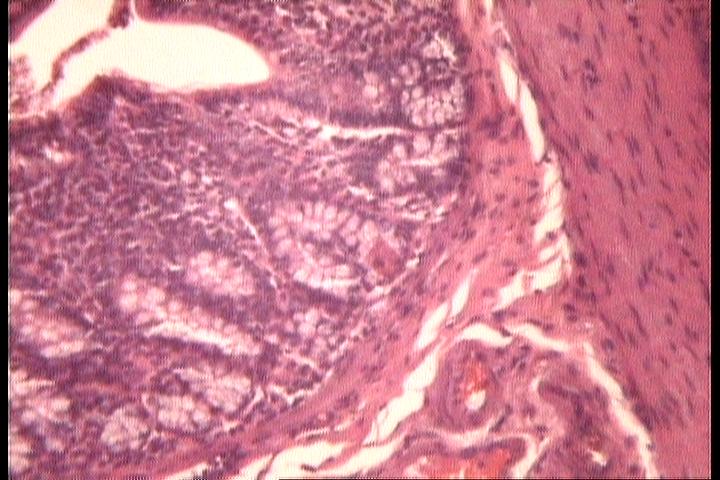
Colon

High dose - male

High dose - female

Ctrl - male

Ctrl - female


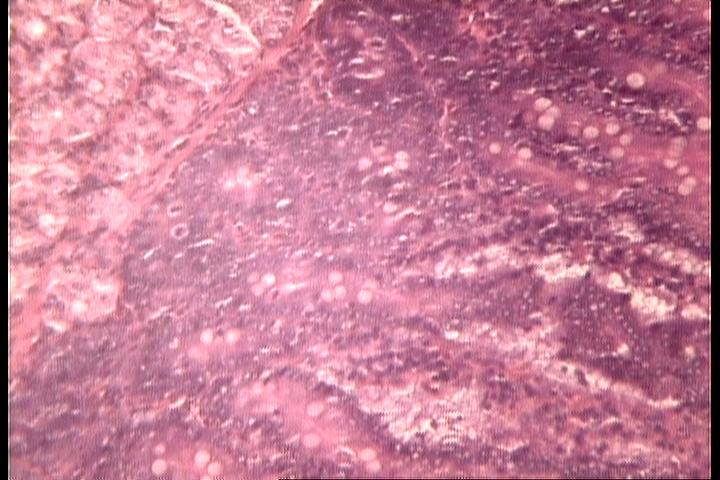

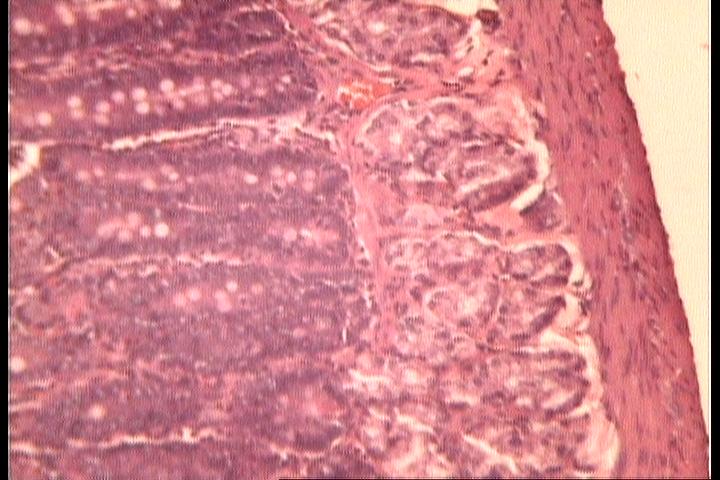

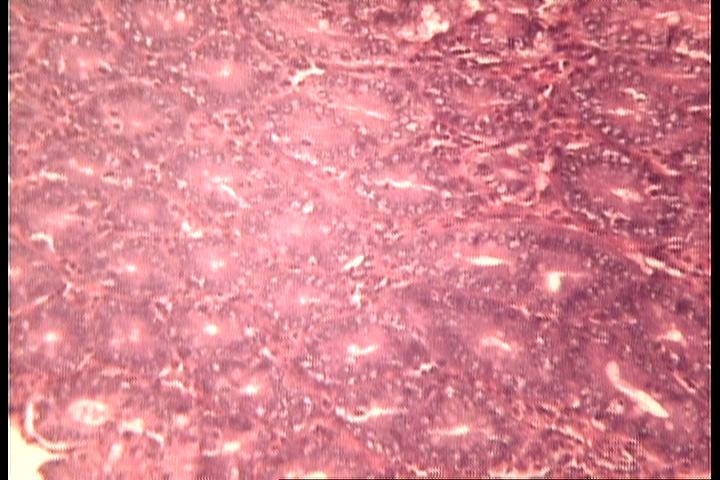

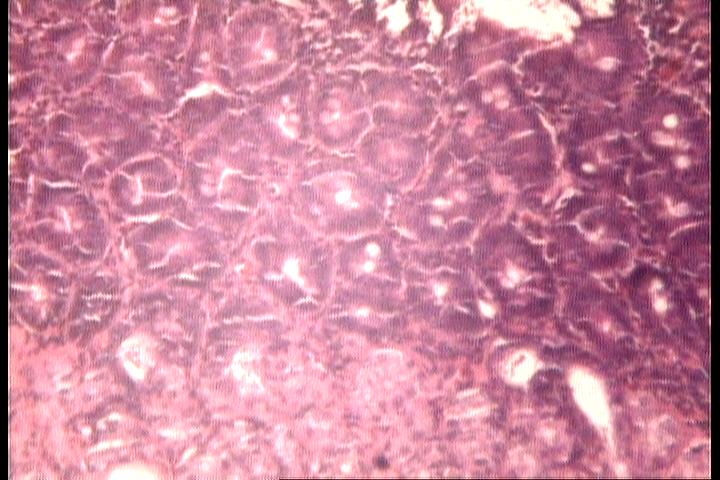
Duodenum

High dose - male

High dose - female

Ctrl - male

Ctrl - female


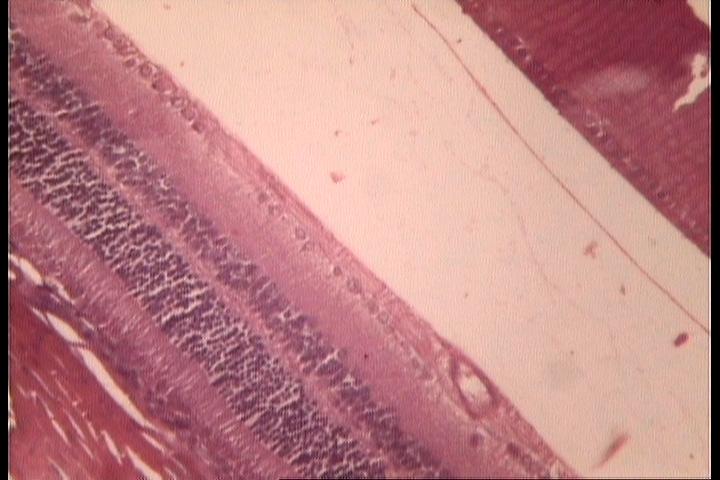

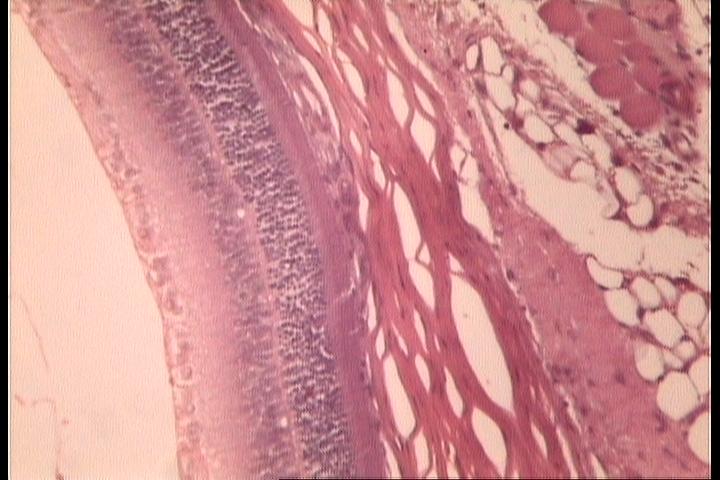

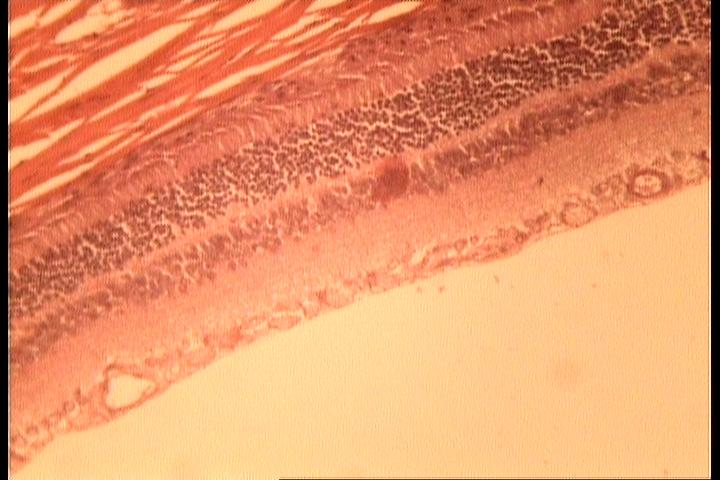

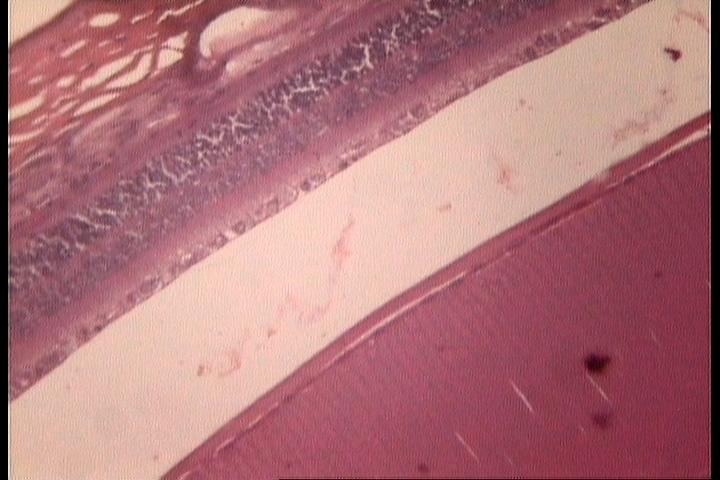
Eye

High dose - male

High dose - female

Ctrl - male

Ctrl - female


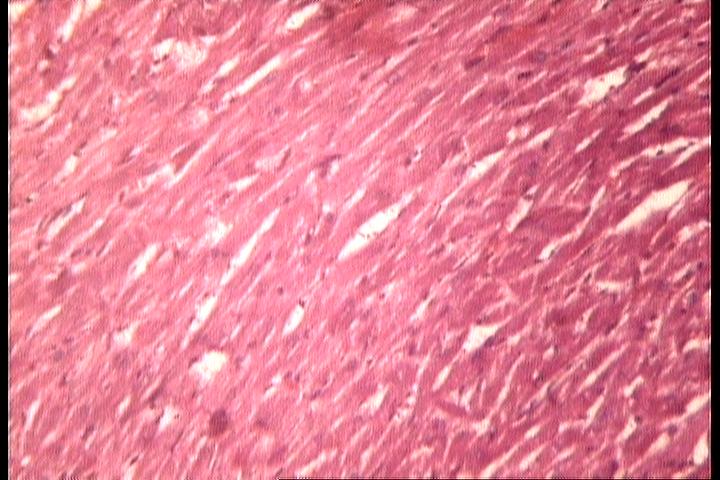

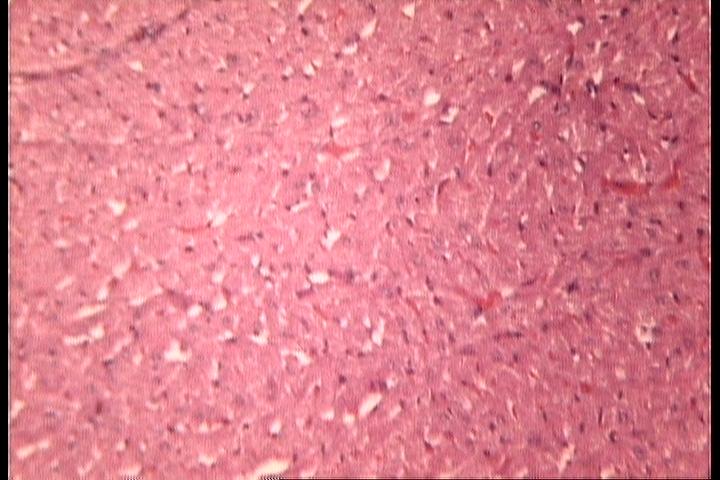

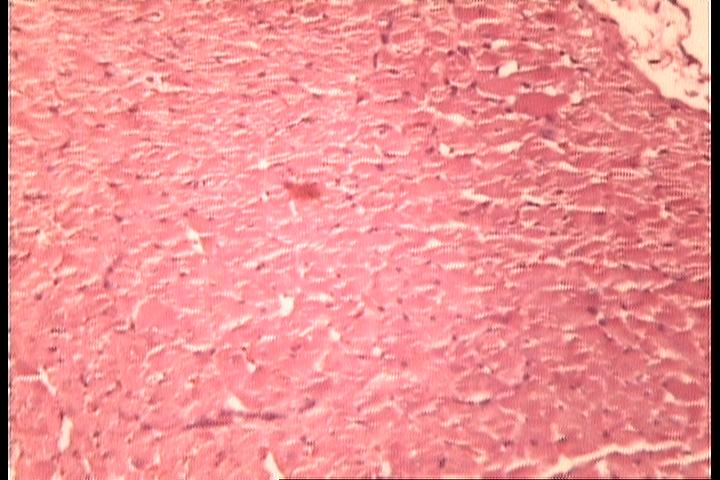

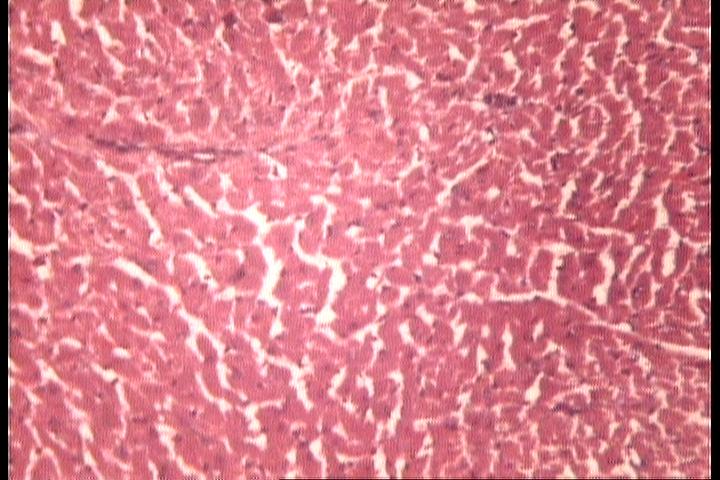
Heart

High dose - male

High dose - female

Ctrl - male

Ctrl - female


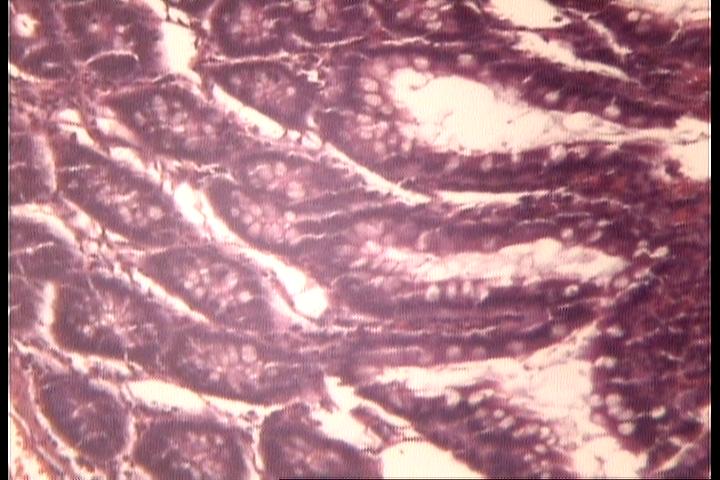

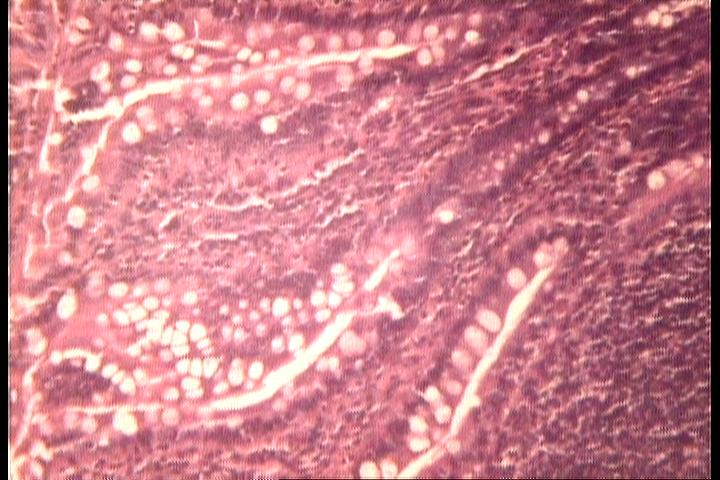

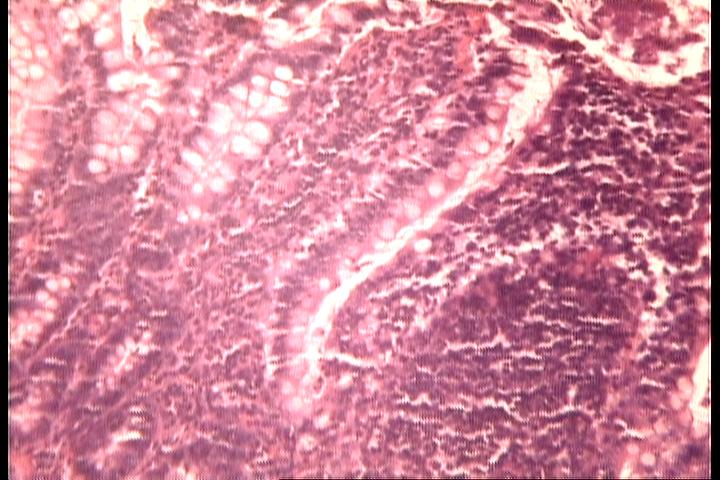

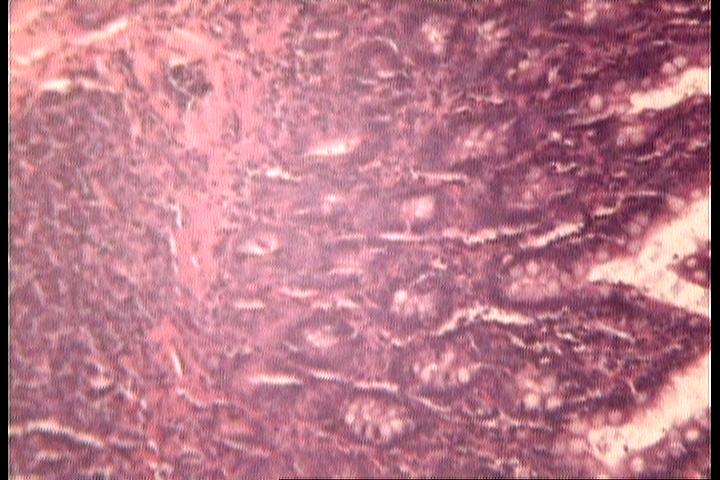
Ileum

High dose - male

High dose - female

Ctrl - male

Ctrl - female


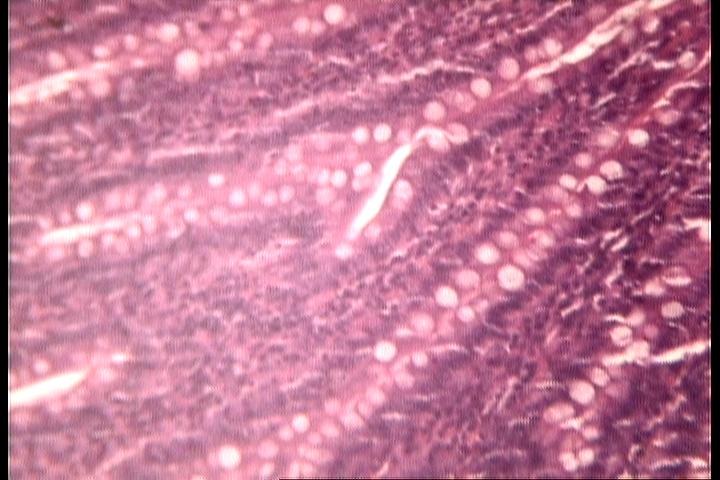

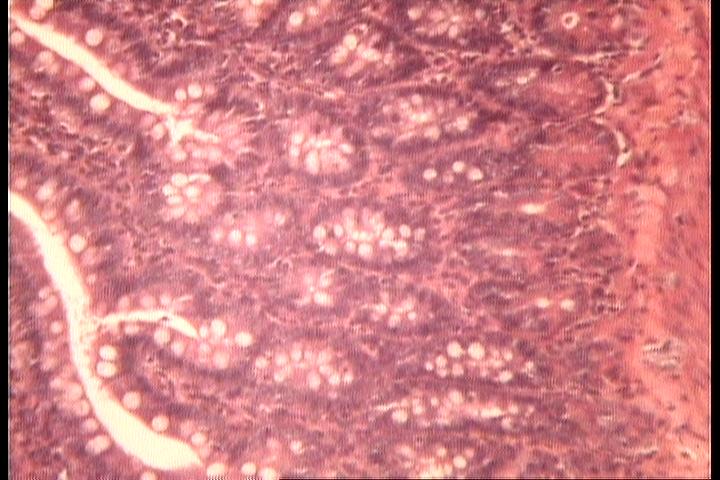

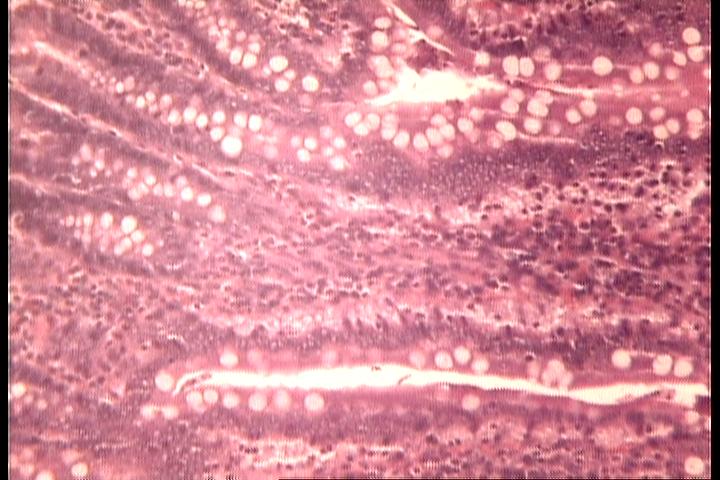

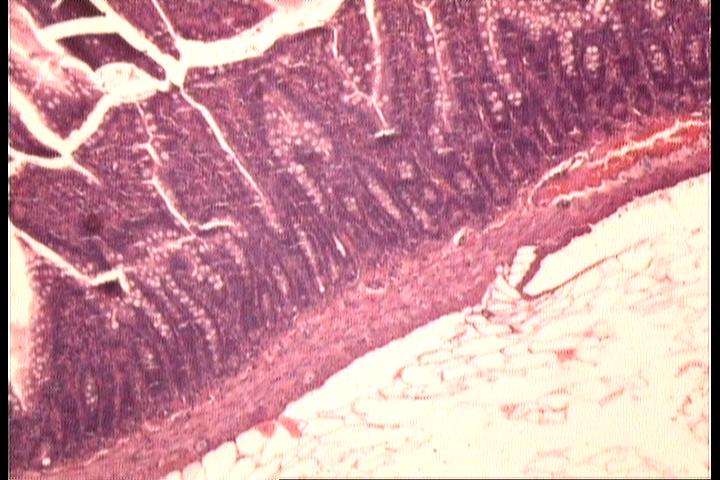
Jejunum

High dose - male

High dose - female

Ctrl - male

Ctrl - female


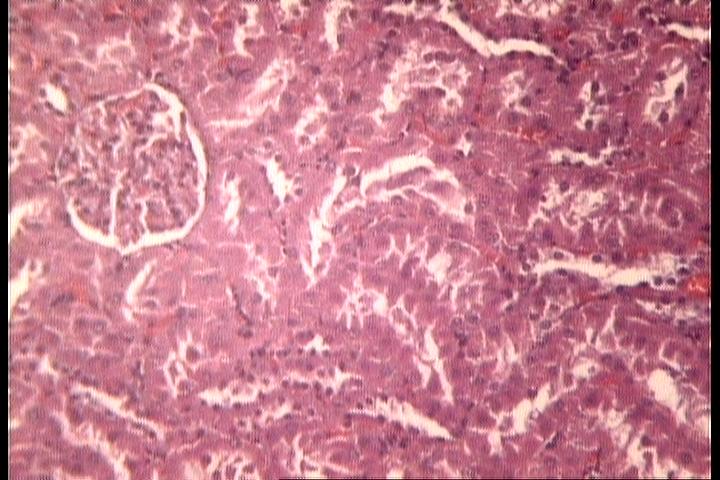

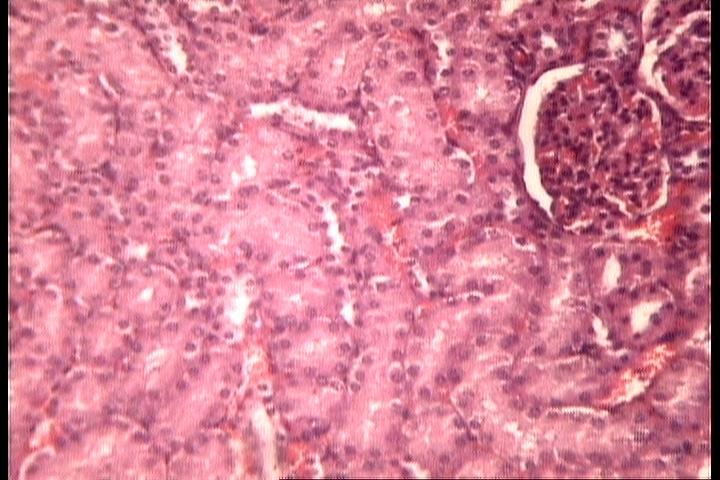

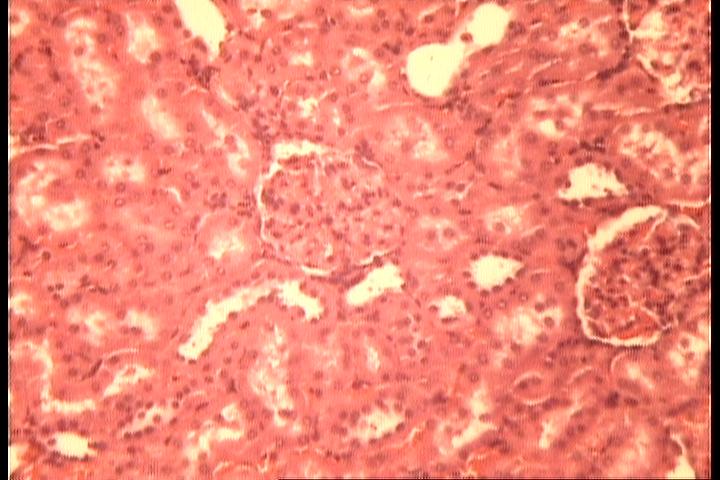

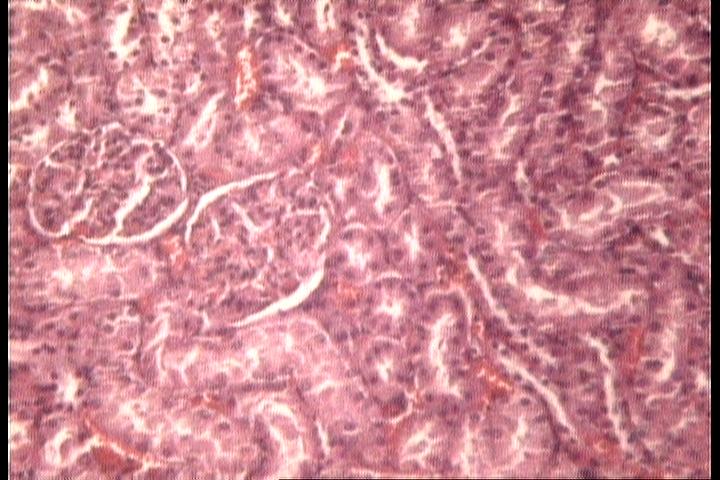
Kidney

High dose - male

High dose - female

Ctrl - male

Ctrl - female


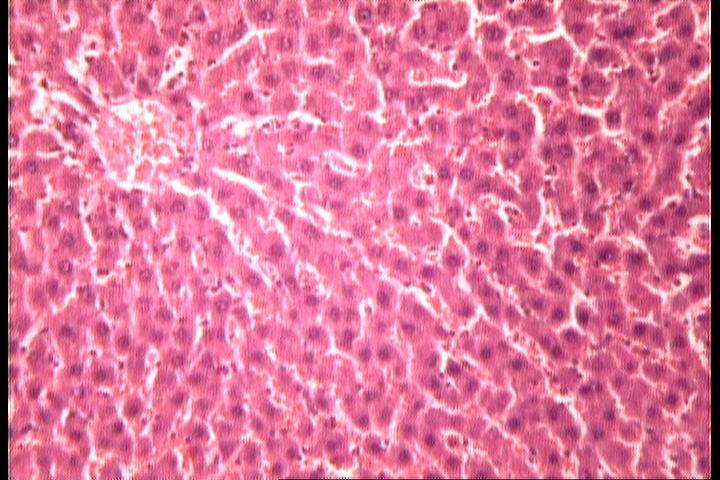

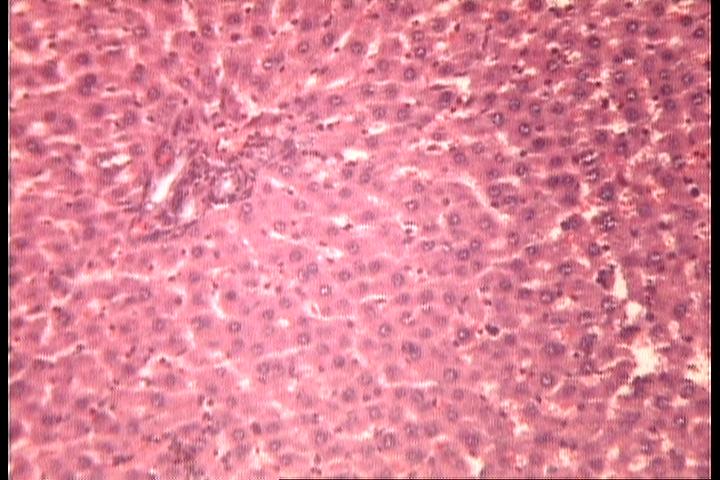

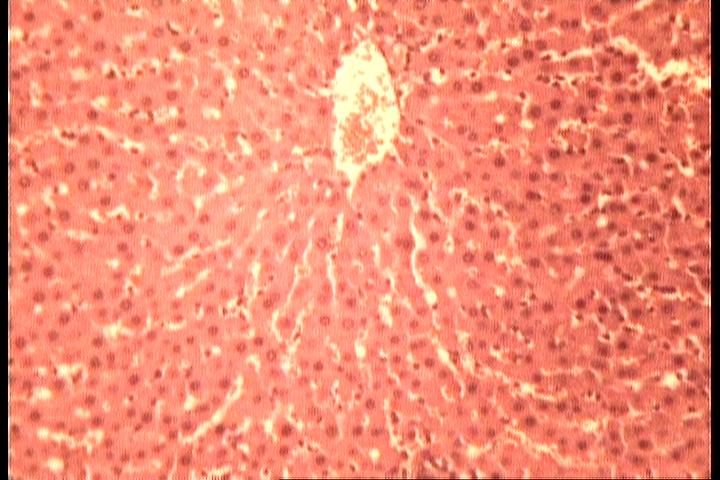

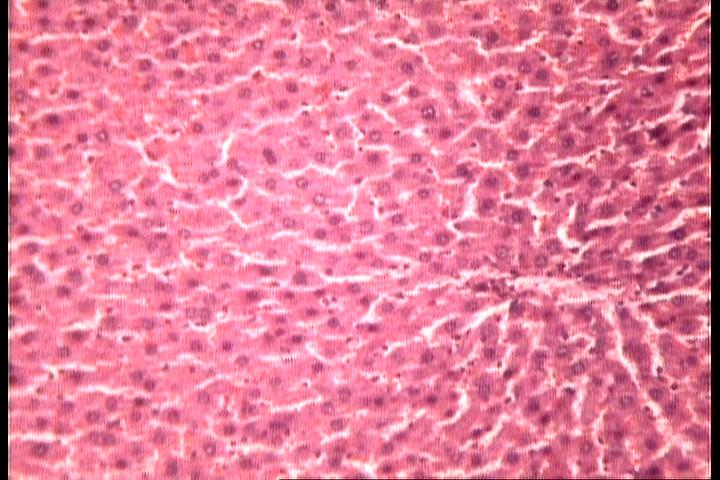
Liver

High dose - male

High dose - female

Ctrl - male

Ctrl - female


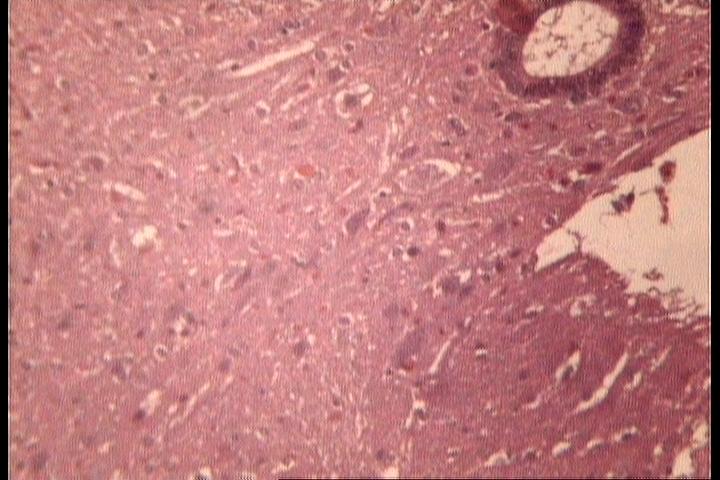

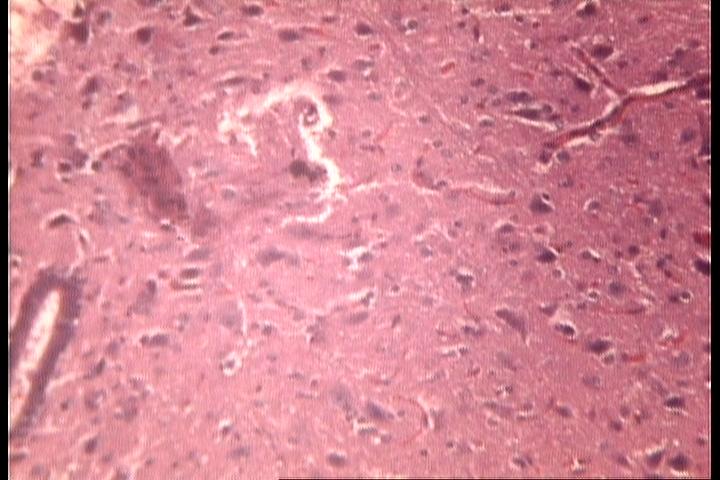

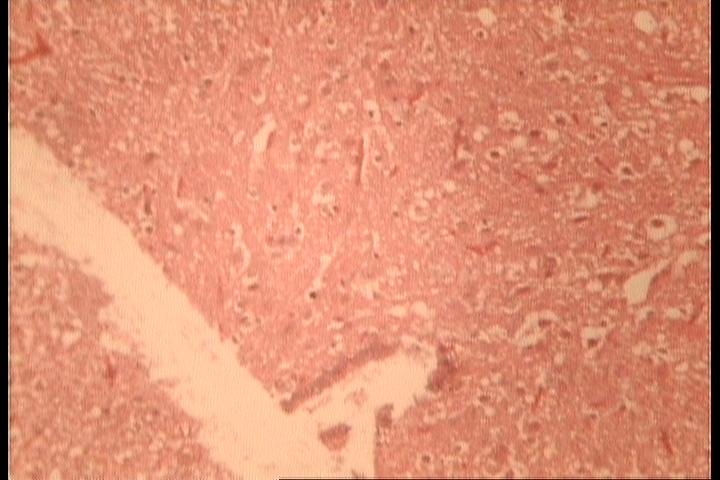

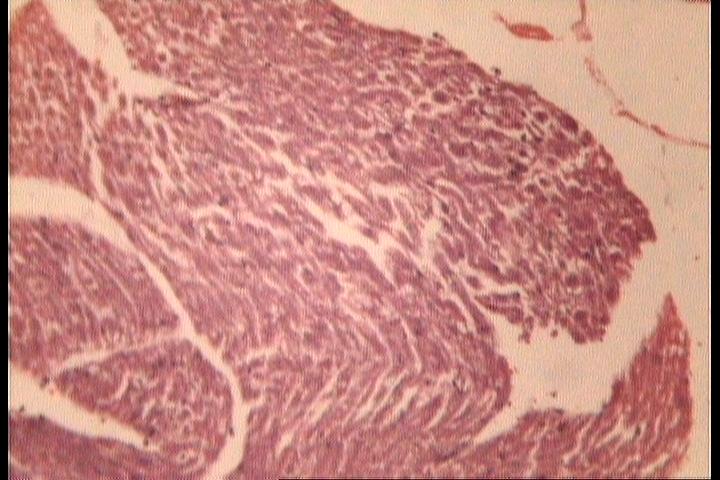
Lumbar Spine

Ctrl - female

High dose - male

High dose - female

Ctrl - male


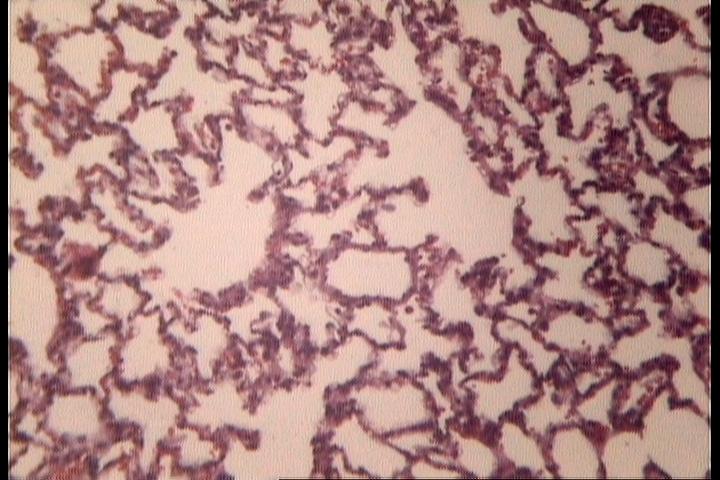

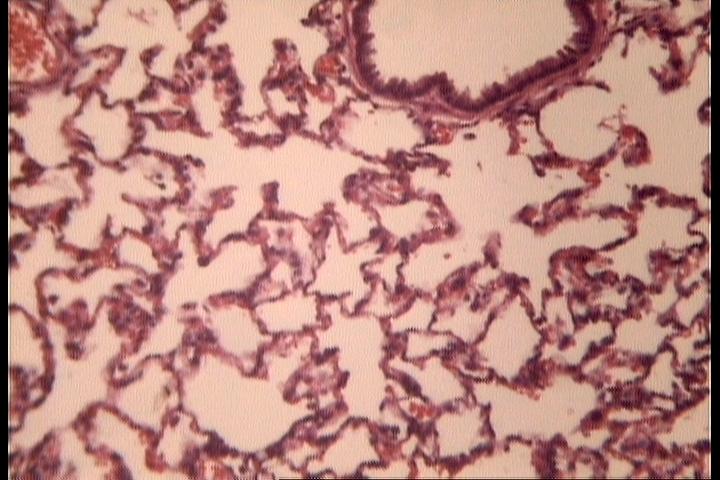

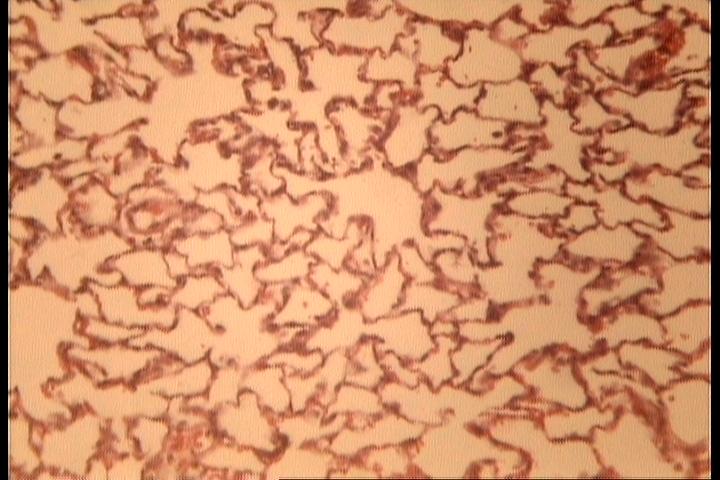

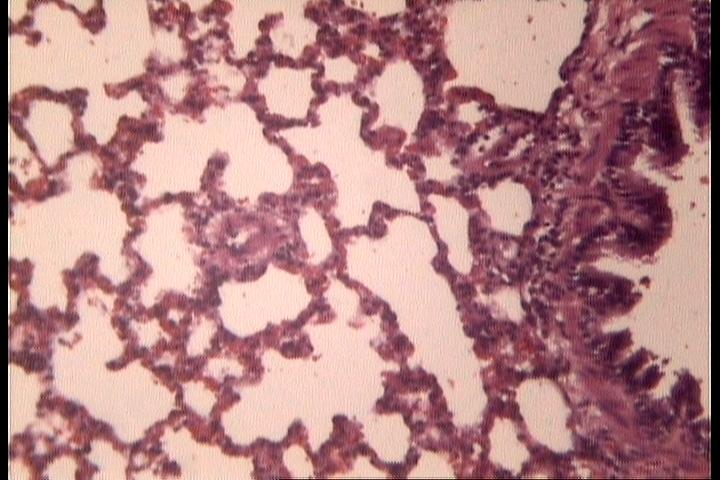
Lung

High dose - male

High dose - female

Ctrl - male

Ctrl - female


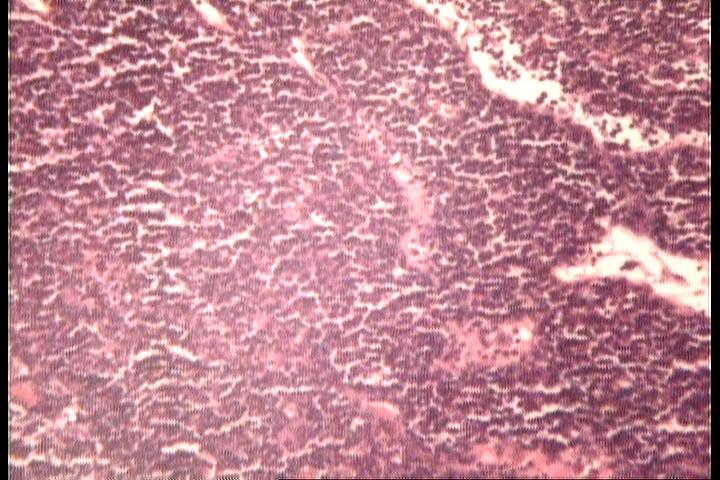

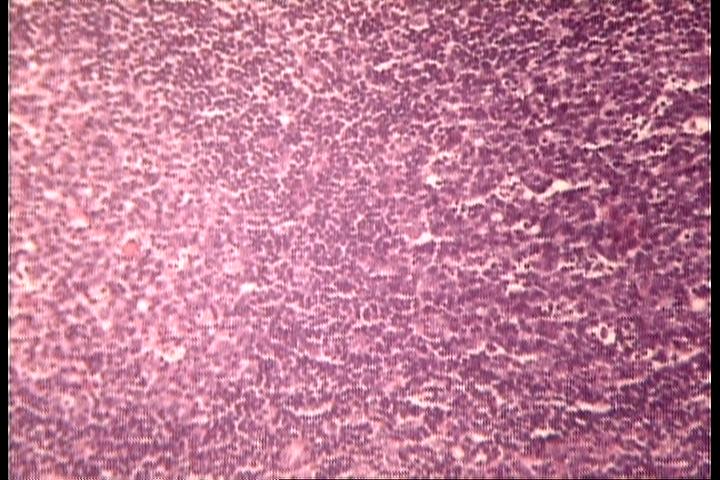

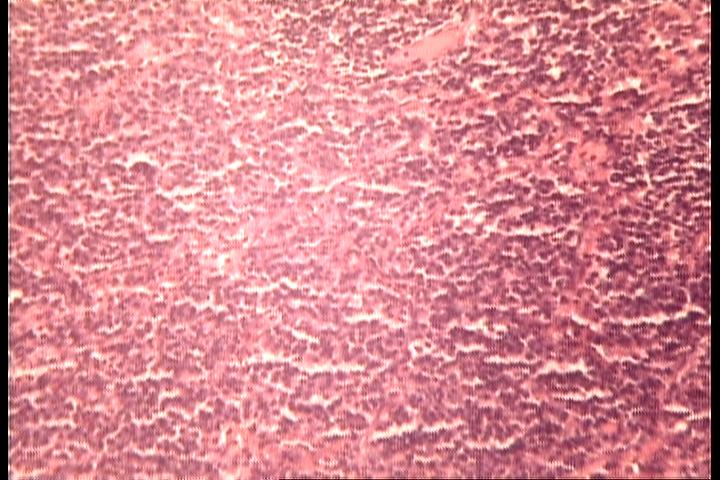

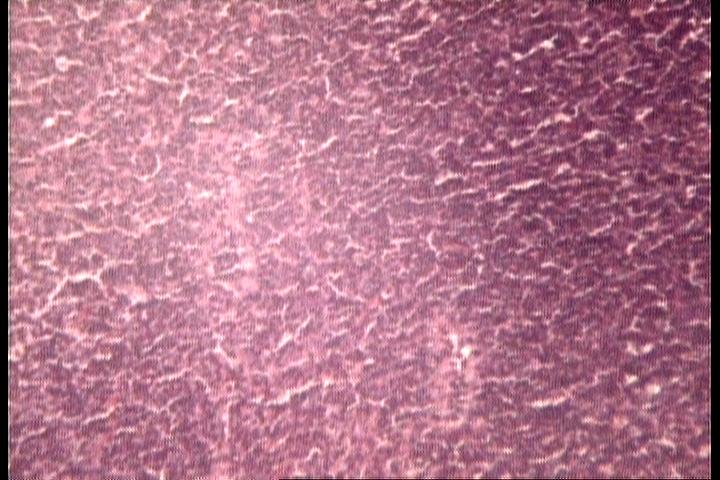
Mesenteric Lymphnode

High dose - male

High dose - female

Ctrl - male

Ctrl - female


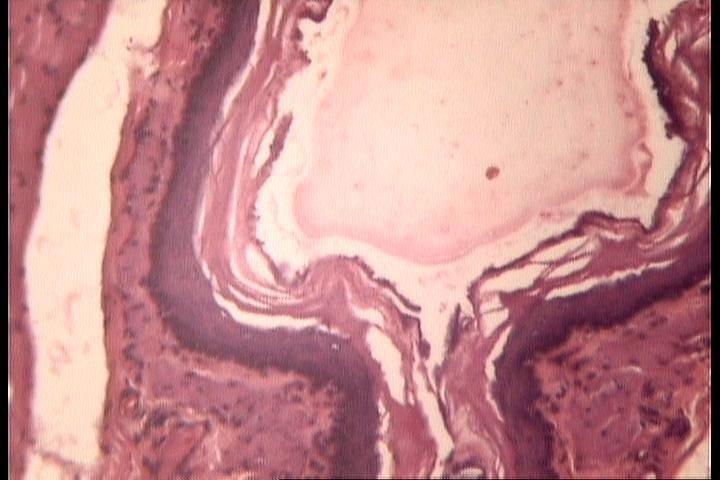

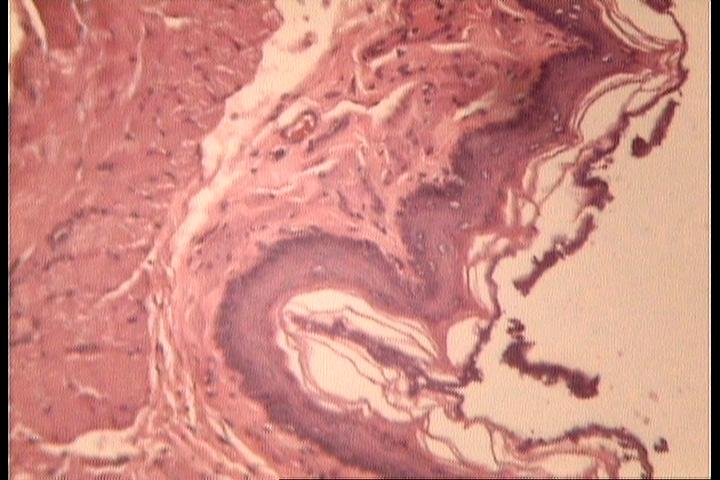

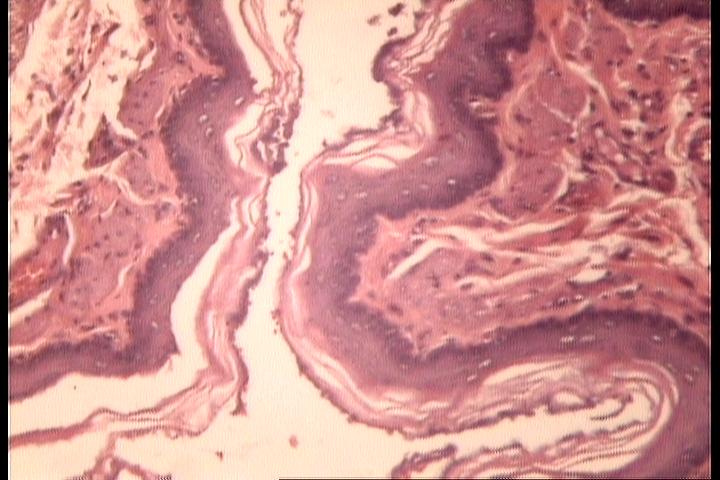

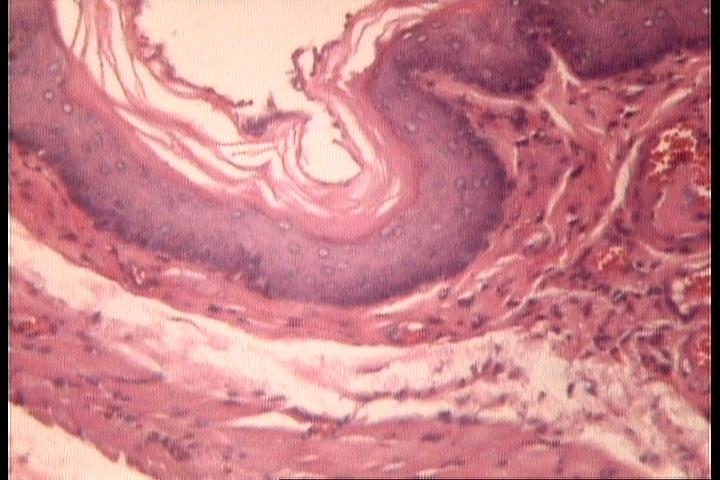
Oesophagus

High dose - male

High dose - female

Ctrl - male

Ctrl - female


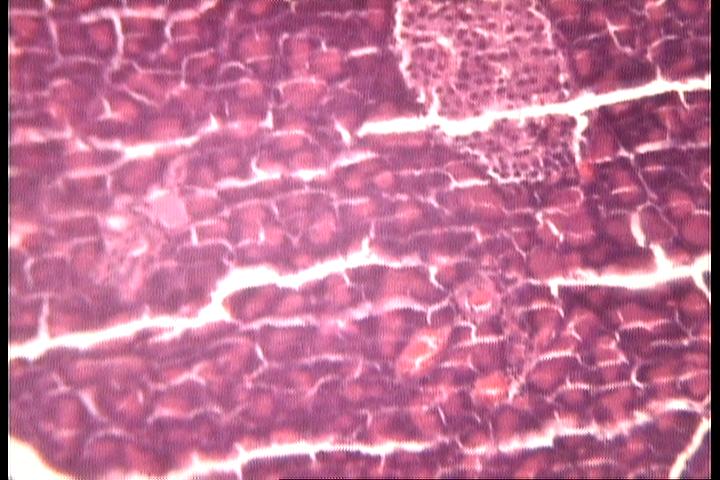

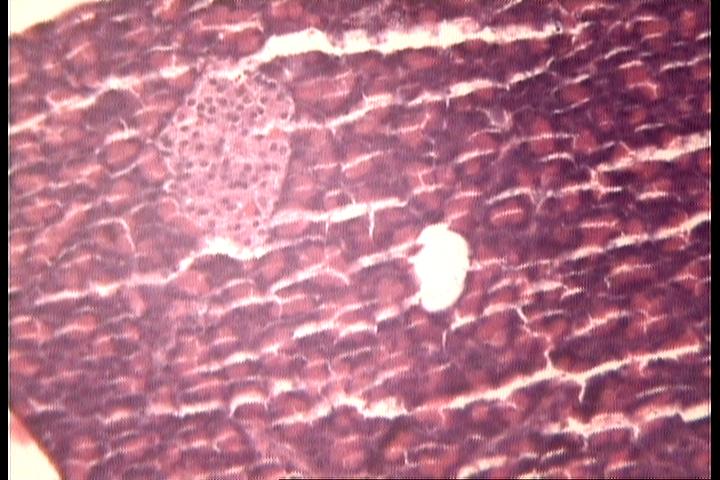

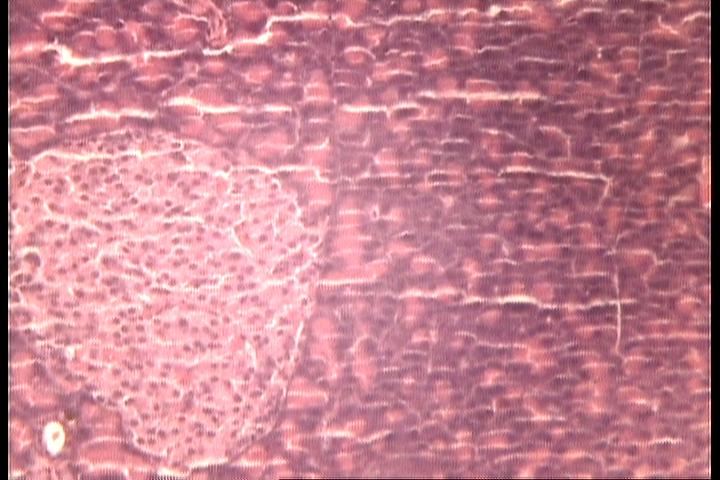

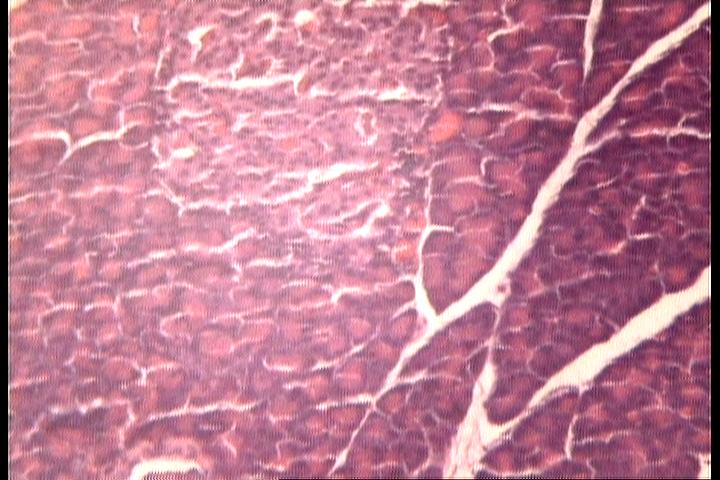
Pancreas

High dose - male

High dose - female

Ctrl - male

Ctrl - female


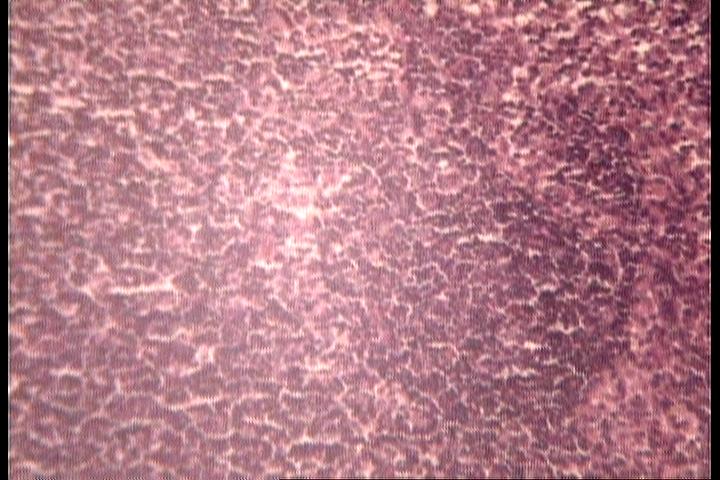

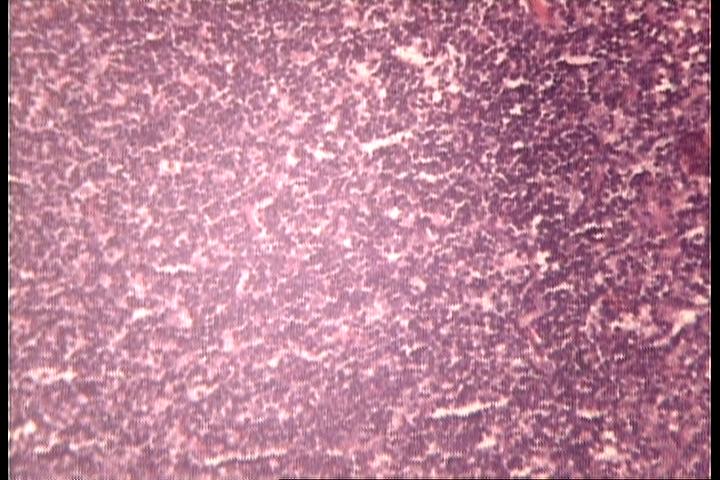

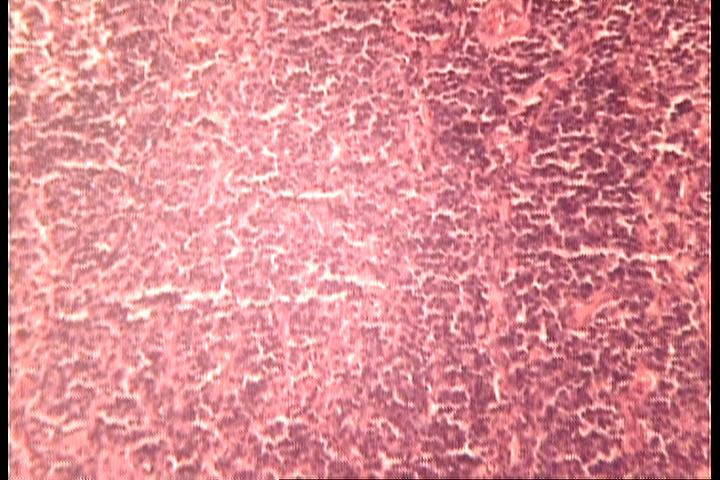

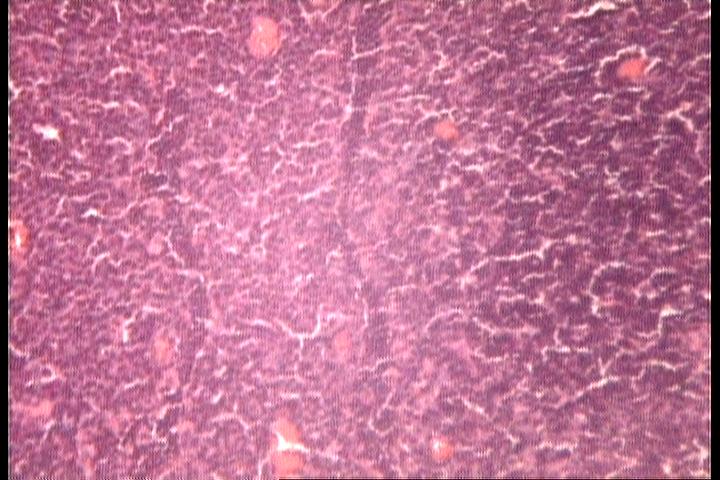
Pharyngeal Lymphnode

High dose - male

High dose - female

Ctrl - male

Ctrl - female


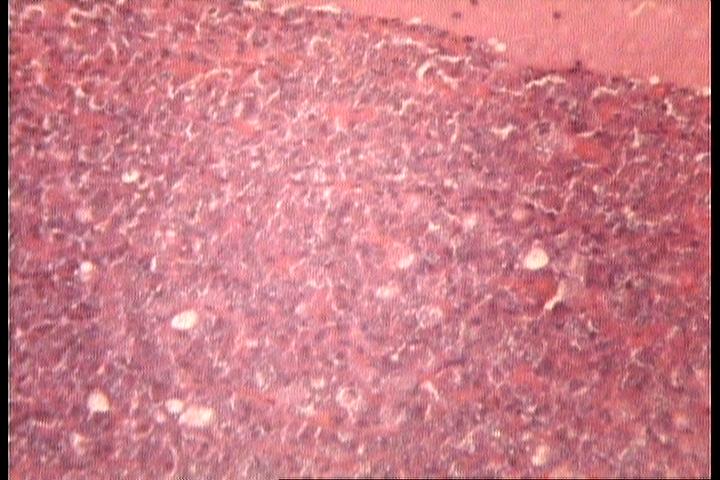

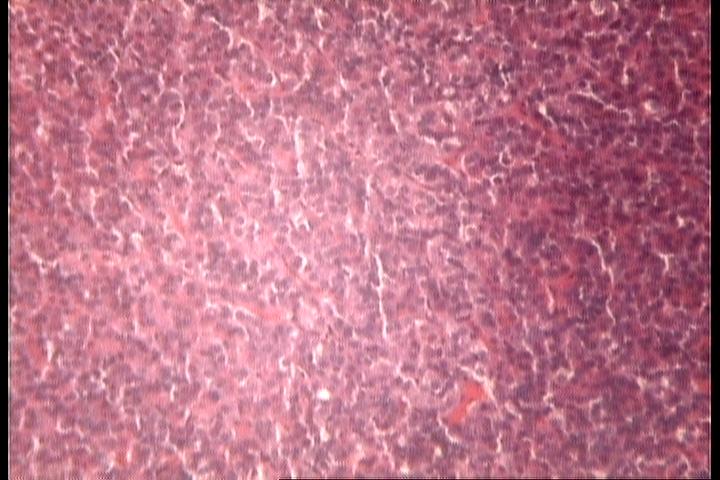

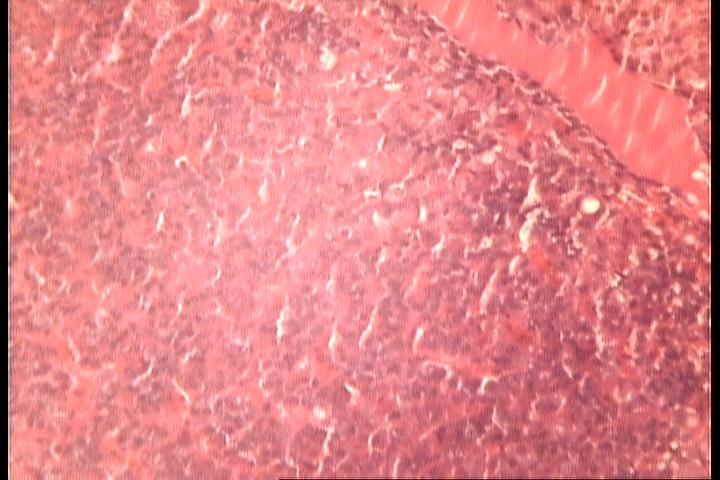

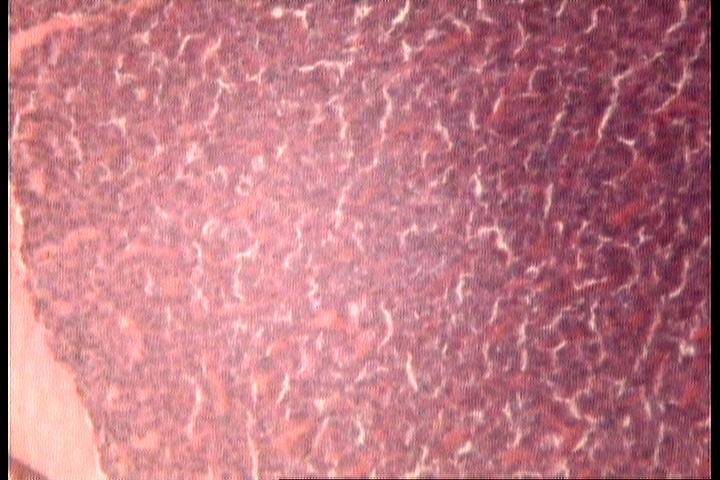
Pitutary

High dose - male

High dose - female

Ctrl - male

Ctrl - female


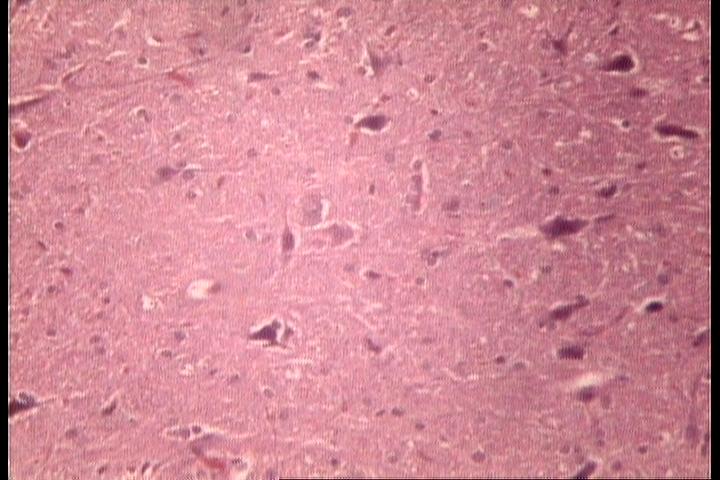

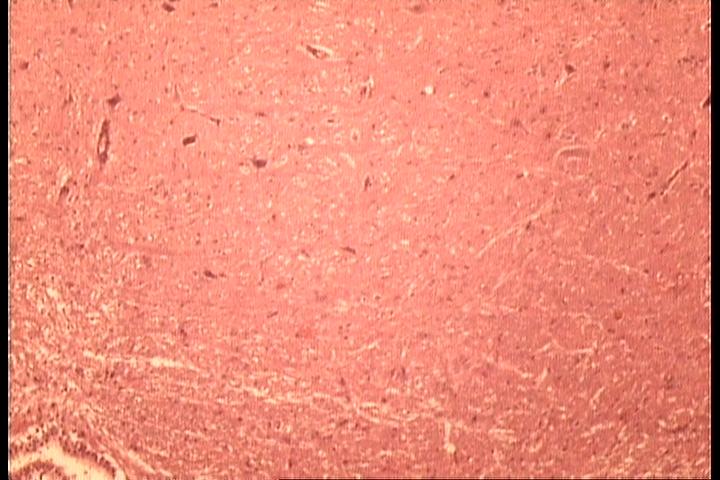

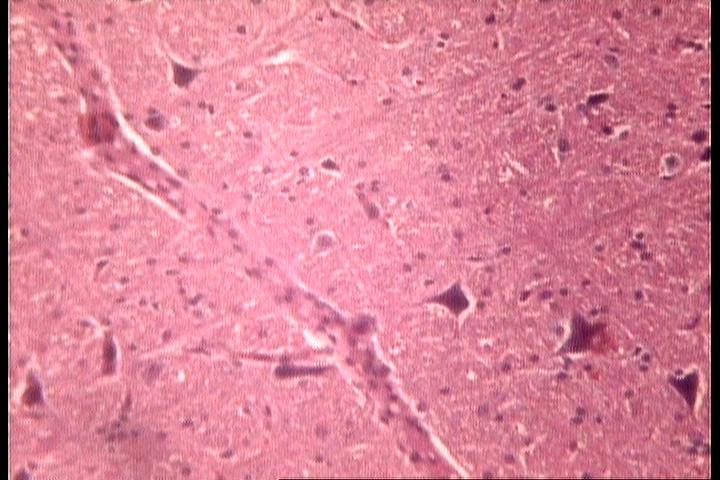
Pons

High dose - male

Ctrl - female


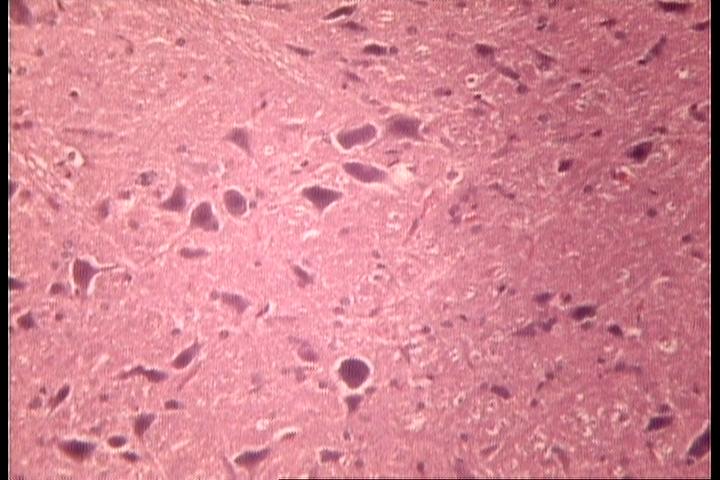


High dose - female

Ctrl - male


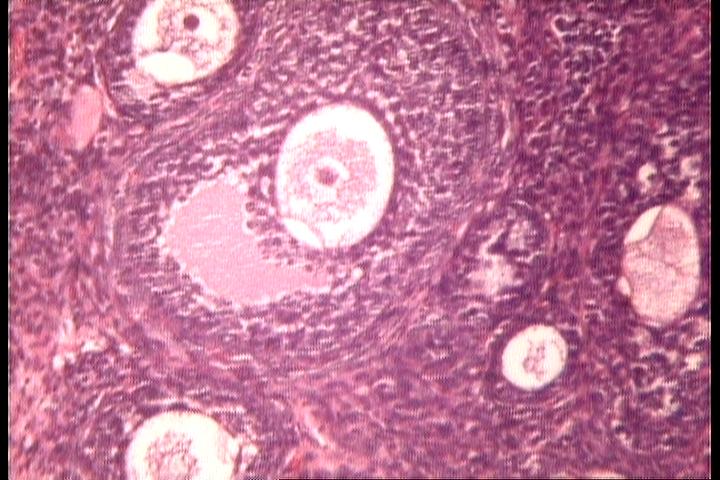

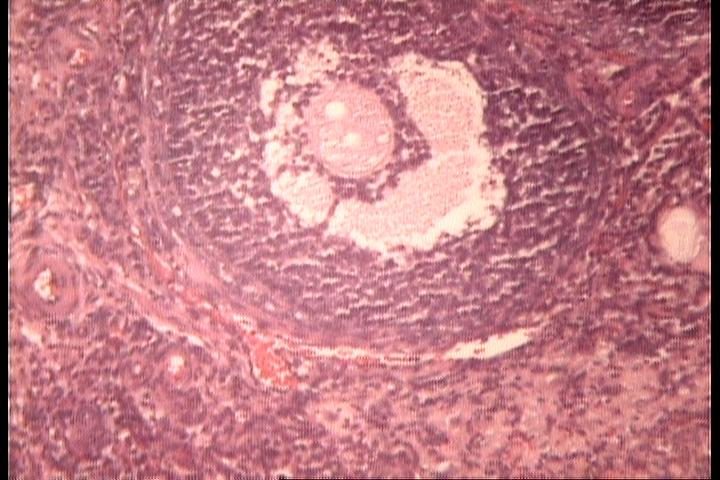
Ovary

Ctrl - female

High dose - female


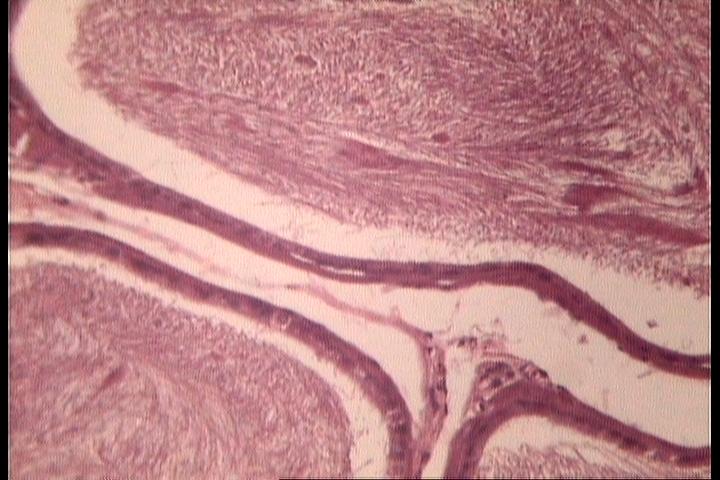

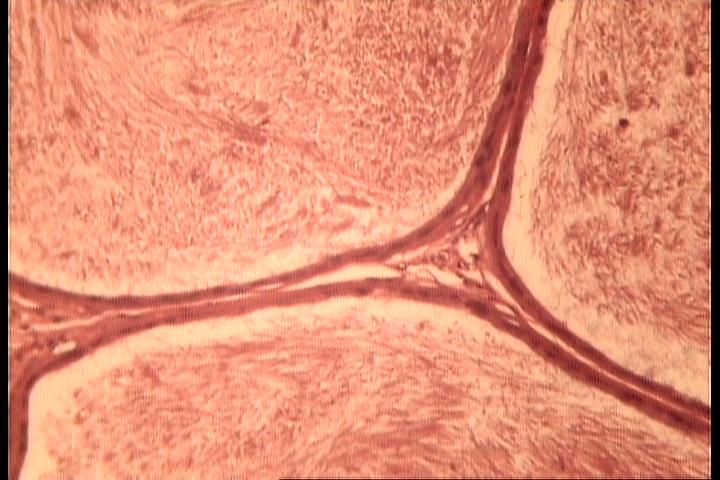
Epidydimis

High dose - male

Ctrl - male


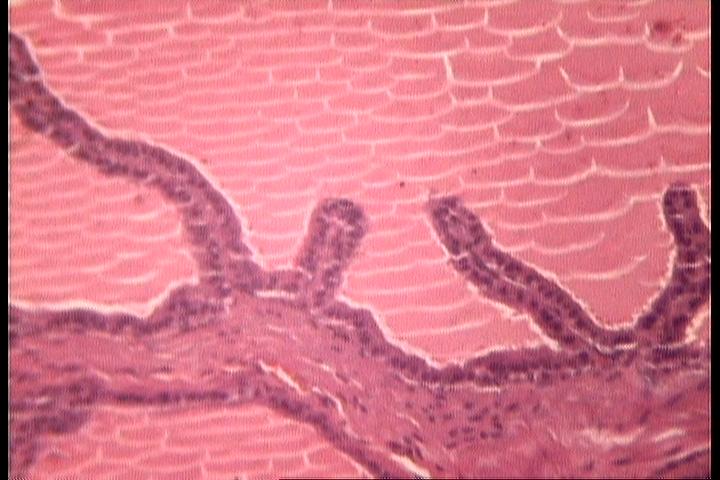

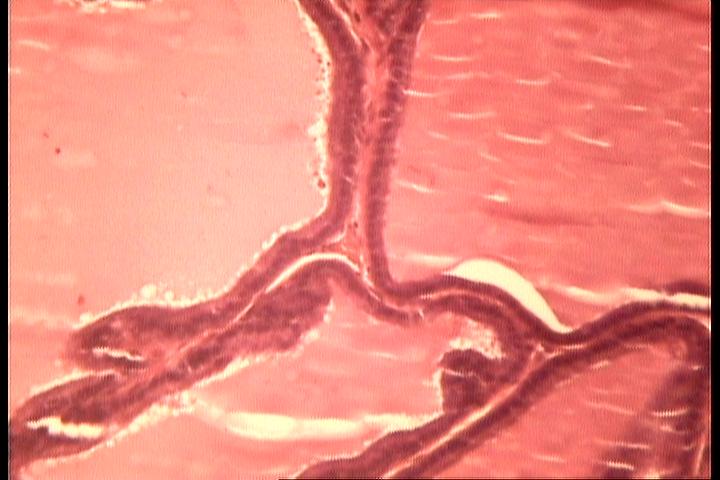
Prostate

High dose - male

Ctrl - male


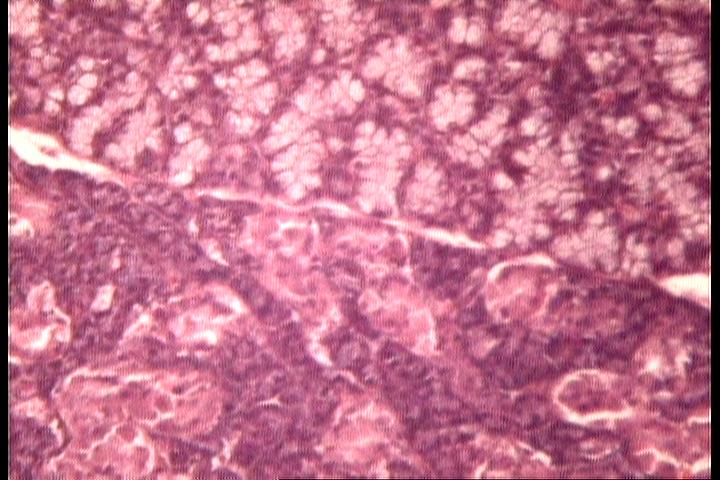

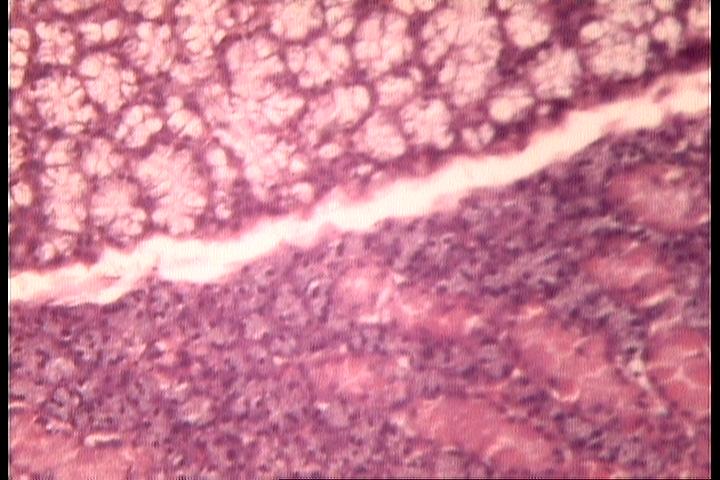
Salivary gland

High dose - female

Ctrl - male

High dose - male

Ctrl - female

Skeletal muscle

High dose - male

High dose - female

Ctrl - male

Ctrl - female

Spleen

Ctrl - male

High dose - male

High dose - female

Ctrl - female

Thymus

High dose - male

High dose - female

Ctrl - male

Ctrl - female

Thyroid

High dose - male

High dose - female

Ctrl - male

Ctrl - female

Trachea

High dose - male

High dose - female

Ctrl - male

Ctrl - female

Urinary bladder

High dose - female

High dose - male

Ctrl - male

Ctrl - female

Uterus

Ctrl - female

High dose - female

Supplementary Figure 1: Histopathology of Ctrl (0 mg/kg) and high dose (1000 mg/kg) male and female rats orally administered with Gutgard for 90days
